# Multi‐Factor Optimization of Nickel Foam Flow Fields: Insights into Structural and Surface Modifications for High‐Performance PEMFCs

**DOI:** 10.1002/advs.202416770

**Published:** 2025-05-24

**Authors:** Siyuan Wu, Chasen Tongsh, Xinmin Ruan, Qing Du, Jae Wan Park, Kui Jiao

**Affiliations:** ^1^ State Key Laboratory of Engines Tianjin University Tianjin 300350 China; ^2^ National Industry‐Education Platform for Energy Storage Tianjin University Tianjin 300350 China; ^3^ Department of Mechanical and Aerospace Engineering University of California Daivs Davis CA 95616 USA

**Keywords:** hydrophobicity, metal foam flow field, pore size, porosity, proton exchange membrane fuel cells, surface treatment

## Abstract

The performance of proton exchange membrane fuel cells (PEMFC) can be significantly influenced by the physical properties of the flow field design. In this study, nickel foam with varying physical parameters—compression (porosity), pore size, hydrophobicity, and anti‐corrosion surface treatments—are systematically investigated to evaluate their influence on PEMFC electrochemical performance, water management, and corrosion resistance. The results reveal that moderate compression (67%), corresponding to a porosity of 85%, offers the optimal balance between electrical conductivity and mass transport, achieving the highest power density of 0.918 W cm^−2^ and a conductivity formation factor 23% higher than uncompressed samples. Excessive compression may cause ligament fractures and occluded cavities, reducing water management capabilities, and increasing parasitic loss and mass transport resistance. Furthermore, smaller pore sizes result in increased concentration losses, primarily due to higher fluid resistance, complex diffusion pathways, and water retention. Hydrophobic surface modification via polytetrafluoroethylene increased water removal efficiency but at the expense of ohmic losses, with excessive loading hindering water transfer and blocking pores. Among various anti‐corrosion coatings, graphene emerged as the optimal choice, providing superior hydrophobicity, corrosion resistance, and electrical conductivity. These findings offer valuable insights for enhancing PEMFC performance and durability.

## Graphical abstract

This article investigates the multi‐factor optimization of nickel foam flow fields in PEMFCs, focusing on compression, porosity, pore size, hydrophobicity, and surface coatings. Through comprehensive experiments and analysis, it reveals how structural and surface modifications influence electrochemical performance, water management, and corrosion resistance, providing guidance for advanced flow field design.

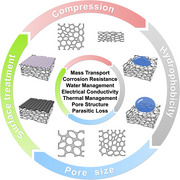

## 1 Introduction

With the growing urgency to address the harmful impacts of carbon emissions on the environment, the global energy system is undergoing a significant transformation from reliance on fossil fuels to the adoption of renewable energy sources. Among these, hydrogen produced from renewable resources is emerging as a promising clean energy carrier. Fuel cells, as an efficient way to utilize hydrogen energy, have gained considerable attention.^[^ ^1^ ^]^ Among the various types of hydrogen fuel cells, proton exchange membrane fuel cells (PEMFCs) stand out due to their high efficiency, low emissions, and rapid start‐up capabilities, making them one of the most promising candidates for widespread application, especially in automotive sector.^[^ ^2^, ^3^ ^]^ However, the large‐scale commercialization of PEMFCs still faces several challenges, particularly in improving performance, durability, and cost‐effectiveness.^[^ ^4^, ^5^ ^]^ One critical aspect that significantly impacts the overall performance of PEMFCs is the design of the flow field. The flow field plays a crucial role in distributing reactant gases, removing by‐products such as water, and ensuring uniform temperature and pressure throughout the fuel cell.^[^ ^6^, ^7^ ^]^ Optimizing the flow field design is essential for enhancing mass transport, minimizing losses, and improving thermal and water management within the fuel cell stack.

Representative configurations of channel‐rib flow fields, such as parallel, serpentine, and interdigitated, and the effects of their geometrical parameters on PEMFC performance have been extensively studied.^[^ ^8^, ^9^, ^10^, ^11^ ^]^ Each flow field design presents unique characteristics that influence gas distribution and water removal, offering distinct advantages and challenges. For instance, serpentine flow fields demonstrate superior water removal capabilities compared to parallel flow fields, leading to enhanced performance. However, the higher pressure drop associated with serpentine designs results in increased parasitic losses, which can reduce overall net power output.^[^ ^12^ ^]^ Similarly, interdigitated flow fields also achieve better mass transport through higher pressure drops, but this benefit must be balanced against the additional parasitic losses.^[^ ^13^ ^]^ In general, the configuration of flow fields has a minimal impact on PEMFC performance at high operating voltages but becomes increasingly significant at lower voltages.^[^ ^14^ ^]^ In serpentine flow fields, increasing the length of straight channel segments and narrowing the channel width enhances reactant gas convection, with longer channels generating greater pressure differences between adjacent channels, thereby improving under‐rib reactant transport.^[^ ^15^ ^]^ Reducing the channel depth also aids in through‐plane transport and water expulsion, with a higher depth‐to‐width ratio further boosting performance.^[^ ^16^, ^17^ ^]^ For parallel flow fields, shortening the channel length and increasing the number of channels improves gas distribution while decreasing the channel‐to‐rib width ratio can significantly enhance performance.^[^ ^18^ ^]^

As advancements in flow field designs continue to address some of the performance limitations of PEMFCs, an emerging alternative to the traditional channel‐rib flow fields has gained attention—porous metal foam flow fields, which has demonstrated several outstanding qualities.^[^ ^19^, ^20^, ^21^, ^22^ ^]^ This design addresses many of the limitations associated with conventional channel‐rib configurations. For instance, while channel‐rib flow fields often suffer from uneven reactant distribution and inefficient water removal, porous metal foam allows for more uniform gas distribution across the entire active area, enhancing mass transfer.^[^ ^23^ ^]^ Additionally, its open‐cell structure significantly reduces the formation of water droplets that can block reactant access to the catalyst layer, thereby improving water management.^[^ ^24^ ^]^ Moreover, the inherent properties of porous metal foam, such as its high specific surface area, mechanical durability, and low pressure drop, have gained significant attention in recent years.^[^ ^25^ ^]^ These features not only contribute to better performance under various operating conditions but also offer the potential for improved fuel cell efficiency and durability.^[^ ^26^ ^]^ Zhang et al.^[^ ^27^ ^]^ developed a 3D model to simulate to performance of PEMFCs using a traditional channel‐rib flow field and metal foam flow field, with a focus on the impact of the metal foam flow field on transport phenomena. The 3D structure of metal foam was reconstructed for this analysis. The results indicated that, in the high current regime, the metal foam flow field significantly enhanced the output power. Moreover, it promoted more uniform distributions of reactants and current density. Li et al.^[^ ^28^ ^]^ developed a scoring system to differentiate water from metal foam ligaments and used an artificial intelligence image processing method to observe the water transport phenomena in metal foam flow fields under varying operating conditions, including relative humidity, temperature, and inlet flow rate. The results demonstrated that metal foam flow fields exhibit high tolerance across a wide range of relative humidity levels, maintaining consistent voltage output even with a 25% variation in humidity. Huo et al.^[^ ^29^ ^]^ developed a cold start model for PEMFC under sub‐zero temperatures, simulating the coupled transport processes of heat, mass, and charge under several sub‐zero conditions and startup modes. The results showed that ice formation occurs more slowly in PEMFCs utilizing metal foam as the cathode flow field during the cold start process, owing to the metal foam's superior water removal capabilities and its ability to distribute gas more uniformly. Additionally, they conducted experiments to evaluate the cold start performance of a PEMFC with a nickel foam flow field.^[^ ^30^ ^]^ The results indicated that the nickel foam flow field outperformed the parallel flow field, primarily due to its enhanced ice storage capacity and improved gas distribution. Furthermore, metal foam fuel cells generated higher output power considering pumping losses. Wu et al.^[^ ^31^ ^]^ systematically investigated the effects of operating conditions on the performance of PEMFC using a metal foam flow field. The results indicated that lower temperatures hinder reaction kinetics, while excessive temperature dehydrates the membrane. Back pressure improves reactant supply but risks flooding. Increasing flow rates aid water removal but raise pumping losses. They also observed that the cathode relative humidity and inlet gas supply had a greater impact on the performance of conventional parallel flow field fuel cells compared to those with metal foam flow fields. Based on these findings, it was recommended to use a thinner gas diffusion layer (GDL) in metal foam fuel cells for improved performance.^[^ ^32^ ^]^ Attributed to the characteristics and functions owned by metal foam that highly overlapped to those of GDL, Tongsh et al.^[^ ^33^ ^]^ successfully assembled a GDL‐less PEMFC. Graphene‐coated nickel foam and ultrathin electrospun carbon nanofiber film were coupled to eliminate conventional channel‐rib flow field and GDL. The thickness of membrane electrode assembly (MEA) was reduced by 90%. The oxygen transport path was shortened by 96%. As a result, the concentration loss was lowered by 88.6%. The GDL‐less PEMFC exhibited superior performance. Its single‐cell volumetric power density was 111% greater than parallel flow field PEMFC. Porous metal foam flow fields are increasingly being considered as a viable option to overcome the challenges faced by traditional flow field designs.

While advancements have been made in exploring the potential of metal foam flow fields as a superior alternative to conventional channel‐rib designs, there remain important aspects that have not been fully investigated. Current research primarily focuses on the overall advantages of metal foam, such as improved gas distribution and water management, yet many of the material‐specific properties of metal foam, including compression, porosity, pore size, hydrophobicity, and surface treatment, have not been systematically analyzed for their direct and combined impact on fuel cell performance. These characteristics play critical roles in determining reactant transport, water management, and durability. In this work, we seek to bridge this gap by conducting a comprehensive study to systematically investigate how variations in compression, porosity, pore size, hydrophobicity, and surface treatment impact mass transport, water management, and overall cell performance. We hope to provide a more detailed understanding of how metal foam can be further optimized to enhance fuel cell operation.

## 2 Experimental Section

### 2.1 Experiment Preparation

#### 2.1.1 PEMFC Single Cell Preparation

**Figure 1:**
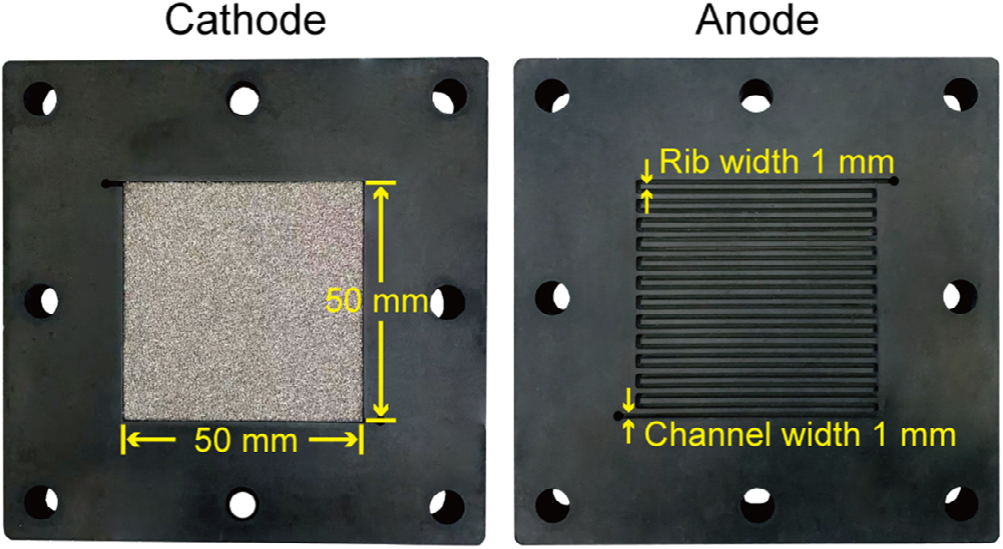
Configuration of the PEMFC single cell.

The PEMFC single cell used in these sets of experiments has an active area of 25 cm^2^, as shown in **Figure** **1**, whose cathode employs nickel foam flow field (with different structural parameters) and anode is serpentine flow field (the flow channel width is 1 mm, the depth is 1 mm, and the channel‐to‐rib ratio is 1). The MEA used is commercially available product with cathode Pt loading of 0.42 mg_Pt_ cm^−2^ and anode Pt loading of 0.07 mg_Pt_ cm^−2^. The membrane and GDL used in this MEA are Gore M775.15 and Toray 060, respectively.

#### 2.1.2 Metal Foam Preparation

In this study, four key physical properties of metal foam, which are compression (porosity), pore size, hydrophobicity, and surface treatment, are focused. Compression is the first parameter been investigated. To isolate the effects of compression, other variables such as pore size and surface treatment are kept constant. Pristine nickel metal foam was selected for this investigation due to its availability and suitability for fuel cell applications. Metal foams with different initial thicknesses but identical structural properties are plastic compressed to a final thickness of 1 mm, which corresponds to the depth of the flow channel. For this study, nickel foam with an 80 PPI pore size is selected. The experiments are divided into three cases: Case A1, Case A2, and Case A3. These cases correspond to the metal foam with initial thicknesses of 2 , 3 , and 5 mm, respectively. After compressed to 1 mm, the compression percentage for case A1, A2, and A3 are 50%, 67%, and 80%, respectively. And the theoretical porosities for case A1, A2, and A3 are 90%, 85%, and 75%, respectively. Detailed parameters for each case are summarized in **Table** **1**.

**Table 1:** Parameters of three Ni foam test samples in the investigation of compression.

Parameters	Case A1	Case A2	Case A3
Pore size	80 PPI	80 PPI	80 PPI
Initial thickness	2 mm	3 mm	5 mm
Final thickness	1 mm	1 mm	1 mm
Original porosity	95%	95%	95%
Compression percentage	50%	67%	80%
Theoretical porosity	90%	85%	75%
Surface treatment	N/A	N/A	N/A

In the second section, three pieces of pristine nickel foam with different pore sizes are utilized to investigate the impacts of pore size on PEMFC performance. Nickel foams with pore sizes of 26 PPI, 40 PPI, and 60 PPI, each with an initial thickness of 3 mm, are selected and compressed to 1 mm. These samples are referred to as Case B1, Case B2, and Case B3, respectively. Hence, the resultant porosity for all cases is 85%. Detailed parameters of three foam samples are provided in **Table** **2**.

**Table 2:** Parameters of three Ni foam test samples in the investigation of pore size.

Parameters	Case B1	Case B2	Case B3
Pore size	26 PPI	40 PPI	60 PPI
Initial thickness	3 mm	3 mm	3 mm
Final thickness	1 mm	1 mm	1 mm
Original porosity	95%	95%	95%
Theoretical porosity	85%	85%	85%
Compression percentage	67%	67%	67%
Surface treatment	N/A	N/A	N/A

Polytetrafluoroethylene (PTFE) is a widely studied material known for its hydrophobicity and corrosion resistance, and it has been extensively applied in various surface modification applications. Owing to its excellent chemical stability, PTFE is chosen in this study to avoid introducing potential uncertainties associated with alternative hydrophobic coating materials, which could affect the performance of PEMFC. However, it is noted that PTFE coating may have a negative impact on the electrical conductivity of metal foam.

To examine the impacts of surface hydrophobicity on the performance of PEMFC, three pieces nickel foam with identical parameters are polytetrafluoroethylene (PTFE) coated with different loadings. The low, moderate, and high PTFE loading foam samples are denoted as Case C1, Case C2, and Case C3, respectively. Details are listed in **Table** **3**.

**Table 3:** Parameters of three Ni foam test samples in the investigation of hydrophobicity.

Parameters	Case C1	Case C2	Case C3
Pore size	40 PPI	40 PPI	40 PPI
Initial thickness	3 mm	3 mm	3 mm
Final thickness	1 mm	1 mm	1 mm
Original porosity	95%	95%	95%
Theoretical porosity	85%	85%	85%
Compression percentage	67%	67%	67%
Surface treatment	Low PTFE	Moderate PTFE	High PTFE

In the last section, the effects of different anti‐corrosive and hydrophobic surface treatments on PEMFC performance are investigated. Three pieces of Ni foam with identical pore size (110 PPI), initial thickness (1.6 mm), and porosity (92% after compression) are utilized. The pristine one and the ones coated with PTFE and graphene are referred to as Case D2, Case D3, and Case D4. A piece of Ni‐Cr alloy foam is selected to be benchmarked against to and referred to as Case D1. However, the commercially available Ni‐Cr alloy is only available with a thickness of 1.2 mm. Therefore, after compression, the resulting porosity of the Ni‐Cr alloy foam is 94%. There is a 2% porosity difference between Case D1 and other cases. The parameters of four foam samples are listed in **Table** **4**.

**Table 4:** Parameters of four Ni/Ni‐Cr foam test samples in the investigation of surface treatment.

Parameters	Case D1	Case D2	Case D3	Case D4
Material	Ni‐Cr alloy	Nickel	Nickel	Nickel
Pore size	110 PPI	110 PPI	110 PPI	110 PPI
Initial thickness	1.2 mm	1.6 mm	1.6 mm	1.6 mm
Final thickness	1 mm	1 mm	1mm	1 mm
Original porosity	95%	95%	95%	95%
Theoretical porosity	94%	92%	92%	92%
Compression percentage	17%	37.5%	37.5%	37.5%
Surface treatment	N/A	N/A	PTFE	Graphene

#### 2.1.3 PTFE Coating

60 wt.% Teflon PTFE DISP 30 is diluted to 5 wt.% using de‐ionized water to better control the PTFE uptake. Before dipping the Ni foam sample into the diluted PTFE dispersion, its initial weight (m_i_) is recorded. Then, the foam sample is immersed in the 5 wt.% PTFE dispersion and gently shaken for couple seconds, then removed and dried in a vacuum drying machine preheated to 80 °C for 15 min. Afterward, the dry weight (m_d_) is recorded and used to calculate PTFE loading using Equation (1) below, once desired PTFE uptake is achieved.

(1) $$\mathrm{PTFE}\mathrm{loading}=\frac{m_{d}-m_{i}}{m_{d}}\times 100\%$$

The Ni foam sample is then placed in a muffle furnace for a two‐step heat treatment: first, at 240 °C for 30 min to remove the surfactant, followed by a second treatment at 380 °C for 30 min to sinter the PTFE. After sintering, the final weight (m_f_) is recorded. The PTFE coating is considered complete if the weight loss, calculated using the Equation (2), is less than 10%. The accurate PTFE loading is then recalculated and recorded using m_f_ and Equation (1).

(2) $$\mathrm{Weight}\mathrm{loss}=\frac{m_{d}-m_{f}}{m_{d}-m_{i}}\times 100\%$$

### 2.2 Characterization Methods

#### 2.2.1 Polarization Curve Measurement

Before conducting polarization curve measurement, a gas tightness test is performed to examine whether the PEMFC is well sealed. Blocking the exhausted pumping, nitrogen is supplied to both anode and cathode until 150 kPa back pressure is reached. Then shut off the gas supply and observe the pressure drop. The PEMFC is considered leak‐free if the pressure drop is less than 5 kPa in 30 min. Then, the PEMFC is supplied with reactant gases and operating at 0.6 V for 15 min, then operating at 0.3 V for 45 min. This operation cycle is repeated 4 times to fully activate the MEA. The polarization curve will be obtained using constant‐current mode. The current increase step size is 0.1 A cm^−2^. The voltage is recorded after the PEMFC stably operating at desired current density for 2 min. The operating conditions for polarization curve measurement are provided in **Table** **5**.

**Table 5:** The fuel cell operating conditions for polarization curve measurement.

Operating condition	Value
PEMFC temperature	70±0.5 °C
Cathode intake flow rate	1.5±(0.04 + 0.8% RD) SLPM
Anode intake flow rate	0.5±(0.02 + 0.8% RD) SLPM
Cathode dew point temperature	64.9 ± 1 °C
Anode dew point temperature	64.9 ± 1 °C
Cathode relative humidity	80 ± 0.8%
Anode relative humidity	80 ± 0.8%
Cathode back pressure (gauge)	0 ± 4.5 kPa
Anode back pressure (gauge)	0 ± 4.5 kPa

#### 2.2.2 Electrochemical Impedance Spectroscopy

Electrochemical impedance spectroscopy (EIS) is conducted using the Zennium pro electrochemical workstation to evaluate the corresponding impedances. EIS test is measured at several representative current density points, until the output voltage reaches 0.3 V. The lower and higher frequency limits are 100 mHz and 100 kHz, respectively. The AC amplitude used during the EIS tests is set to 5% of the PEMFC operating current at the time of testing.

#### 2.2.3 Contact Angle Measurement

**Figure 2:**
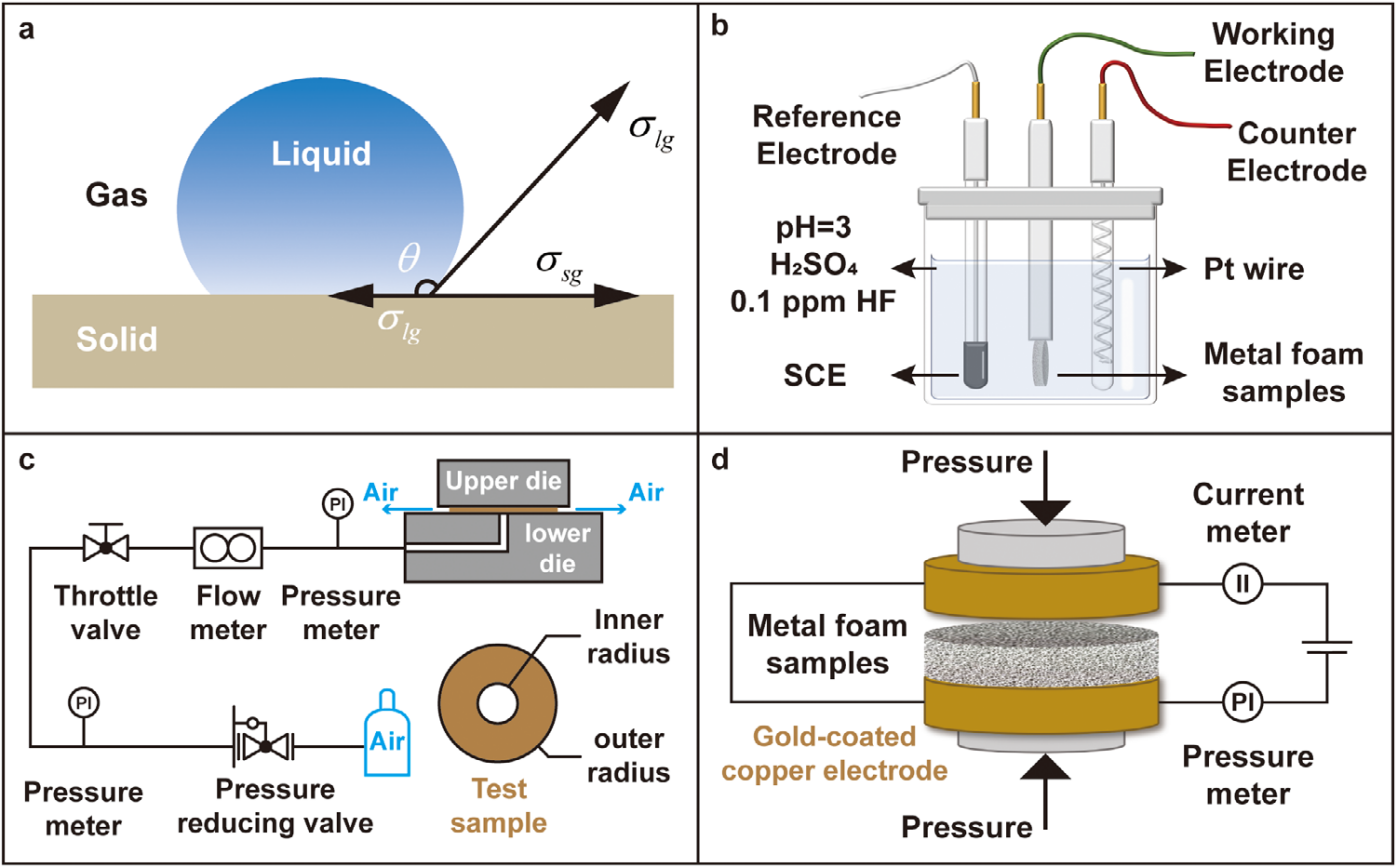
Illustration of a) contact angle; b) three‐electrode corrosion current measurement system; c) metal foam in‐plane gas permeability test system; and d) metal foam through‐plane electrical resistance test system.

The hydrophobicity of the metal foam samples, each with varying materials and surface treatments, is assessed using water contact angle measurements. The contact angle is defined as the angle formed at the intersection of a liquid‐vapor interface and a solid surface, typically measured from the liquid side, as shown in **Figure** **2**a. The solid surface is defined hydrophobic if the value of the contact angle (θ) is greater than 90°, and is defined hydrophilic if the value of the contact angle is less than 90°.^[^ ^34^ ^]^ The contact angle is associated with the surface tension of the liquid (σ_*Ig*_), the interfacial tension (σ_*sI*_) between liquid and solid, and the surface free energy (σ_*sg*_) of the solid, by Young's Equation (Equation (3)). The contact angle of metal foam samples is measured and fitted using the Krüss DSA100 drop‐shape analyzer.

(3) $$\sigma_{sg}=\sigma_{sl}+\sigma_{lg}\cdot \cos\theta$$

#### 2.2.4 Corrosion Current Measurement

As shown in Figure 2b, a three‐electrode system is employed, with the working electrode being the testing sample, the reference electrode being Saturated Calomel Electrode (SCE), and the counter electrode being a platinum wire. The measurement is conducted in a mixture of hydrofluoric acid and sulfuric acid solution with a pH of 3 and F^−^ concentration of 0.1 mg L^−1^ at PEMFC operating temperature of 70 °C. The test sample is fully immersed in the solution and allowed to stabilize for at least 30 min to obtain its open‐circuit voltage.

##### Potentiodynamic Test

An electrochemical workstation is used to perform a linear potential sweep from a negative potential to a positive potential at a sweep rate of 2 mV s^−1^. The sweep range spans from −500  to +1400 mV versus SCE. The measured curve is linearly fitted at its cathodic and anodic Tafel regions using the Tafel extrapolation method, and the lines are extrapolated to their intersection to determine the corrosion potential and corrosion current. These results are then compared and analyzed. The corrosion current at 0.6 V versus SCE is also commonly used for convenience.

##### Potentiostatic Test

An electrochemical workstation is used to applied a constant potential of 600 mV versus SCE and hold for 21600 s. The measured polarization curve is processed by taking the average corrosion current over the final 5 min as the steady‐state corrosion current under constant potential, which is used to characterize the long‐term corrosion behavior.

The corrosion current density is then calculated using Equation (4) below.

(4) $$I_{corr}=\frac{I}{S}$$

where, *I_corr_* is the corrosion current density (µA cm^−2^), *I* is the corrosion current (µA), and *S* is the effective test surface area (cm^2^).

#### 2.2.5 Porosity Calculation

The theoretical porosity of compressed Ni foam samples can be calculated using Equation (5) below:

(5) $$\begin{array}{ccc} \varepsilon_{th} & = & \frac{V_{pore}}{V_{comp}}\times 100\%=\frac{V_{comp}-V_{solid}}{V_{comp}}\times 100\%\\ & & =\frac{V_{comp}-\left(1-\varepsilon_{i}\right)\times V_{i}}{V_{comp}}\times 100\% \end{array}$$

where, ε_*th*_is the theoretical porosity of the compressed Ni foam sample, *V_pore_* is the total volume of pores (mm^3^), *V_comp_* is the total volume of the compressed Ni foam sample (mm^3^), *V_solid_* is the total volume of foam ligaments (mm^3^), ε_*i*_ and *V_i_* are the initial porosity and initial volume of Ni foam sample before compression, respectively.

#### 2.2.6 Parasitic Loss Calculation

The parasitic loss can be calculated by the Equation (6) below:

(6) $$P_{parasitic}=\frac{\Delta P\cdot Q}{60\cdot \eta }$$

where *P _parasitic_* is the parasitic loss caused by the air compressor (W); *ΔP* is the pressure drop (kPa); *Q* is the intake flow rate (SLPM); *η* is the air compressor efficiency. Equation (7) is used to calculate the parasitic loss ratio:

(7) $$\eta_{loss}=\frac{P_{parasitic}}{P_{PEMFC}}\times 100\%$$

where, *η_loss_* is the parasitic loss ratio (%); *P_PEMFC_* is the peak power of the PEMFC (W).

#### 2.2.7 Limiting Current Density Measurement

The limiting current density is measured by supplying a gas mixture consisting of 1% dry molar fraction oxygen ($x_{O_{2}}$) in nitrogen (99.999% purity) to the cathode. The anode is supplied with hydrogen (99.999% purity). The detailed test conditions are listed in **Table** **6**. A polarization curve is obtained, and the current density at 0.1 V is defined as the limiting current density (*i*_lim_). The oxygen transport resistance is then calculated based on the limiting current density. At the limiting current condition, for a specific oxygen molar fraction and cathode pressure, the oxygen transportation resistance (*R_t_*) can be calculated using Equation (8).

(8) $$R_{t}=\frac{4Fx_{O_{2}}\left(p_{ch}-p_{w}\right)}{RT}\times \frac{1}{i_{\lim}}$$

where, *p_ch_* is the absolute pressure in the cathode flow field (kPa), *p_w_* is the water vapor partial pressure in the cathode gas mixture (kPa), *R* is the universal gas constant (8.314 J mol^−1^ K^−1^), *F* is the Faraday's constant (96 485 C mol^−1^), *T* is the cell operating temperature (K), and $x_{O_{2}}$ is the molar fraction of oxygen in the cathode inlet gas.

**Table 6:** Testing conditions of limiting current density measurement.

Operating condition	Value
PEMFC temperature	343 K
Cathode air intake flow rate	0.07 SLPM
Cathode nitrogen intake flow rate	1.43 SLPM
Dry mole fraction of oxygen	0.98%
Anode intake flow rate	0.5 SLPM
Cathode dew point temperature	343 K
Anode dew point temperature	343 K
Cathode relative humidity	100%
Anode relative humidity	100%
Partial pressure of water vapor	31.16 kPa

#### 2.2.8 In‐Plane Gas Permeability Measurement

The metal foam sample is cut into a concentric ring with an inner radius *R*_1_ = 6*mm* and an outer radius *R*_2_ = 20*mm*. The in‐plane gas permeability test system is illustrated in Figure 2c. The sample is placed between the upper and lower dies of the testing apparatus, ensuring full surface coverage by the dies. A pressure of 1 MPa is applied to the dies. Dry air is supplied, and the throttle valve is adjusted to achieve a pressure difference of 0.01 kPa, which is maintained for 15 s. The corresponding volumetric air flow rate is then recorded. The in‐plane gas permeability of the metal foam, *V_in_*, can be calculated using Equation (9).

(9) Vin=60×100000×Vs×lnR2R2R1R12×3.14×d×Ps

where, *V_in_* is the in‐plane gas permeability of the sample (mL mm cm^−2^ h^−1^ Pa^−1^), *V_s_* is the volumetric flow rate of air through the sample under the given pressure difference (mL min^−1^), *R*_1_ is the inner radius of the metal foam sample (mm), *R*_2_ is the outer radius of the metal foam sample (mm), *P_s_* is the pressure difference (Pa), and *d* is the sample thickness (µm)

#### 2.2.9 Through‐Plane Resistance Measurement

The through‐plane resistivity test system is illustrated in Figure 2d. The metal foam sample is placed between two gold‐coated copper electrodes. A pressure of 200 kPa is applied to the electrodes and held for 15 s to stabilize the contact. The resistance *R_m_* is obtained by recording the current and voltage values during the test. The through‐plane resistivity ρ of the sample can then be calculated using the following Equation (10).

(10) $$\rho =10^{4}\times \frac{R_{m}S\varepsilon -R_{c}}{d}$$

where, ρ is the metal foam through‐plane resistivity (mΩ cm), *R_m_* is the measured resistance (mΩ), *S* is contact area of the metal foam sample (cm^2^), ε is the porosity of the metal foam sample, *d* is the thickness of the metal foam sample (µm), and since gold‐coated copper electrodes are used, the bulk resistance of the electrodes and the contact resistance between the sample and the electrodes (*R_c_*) is assumed to be negligible.

## 3 Results and Discussion

### 3.1 Compression

**Figure 3:**
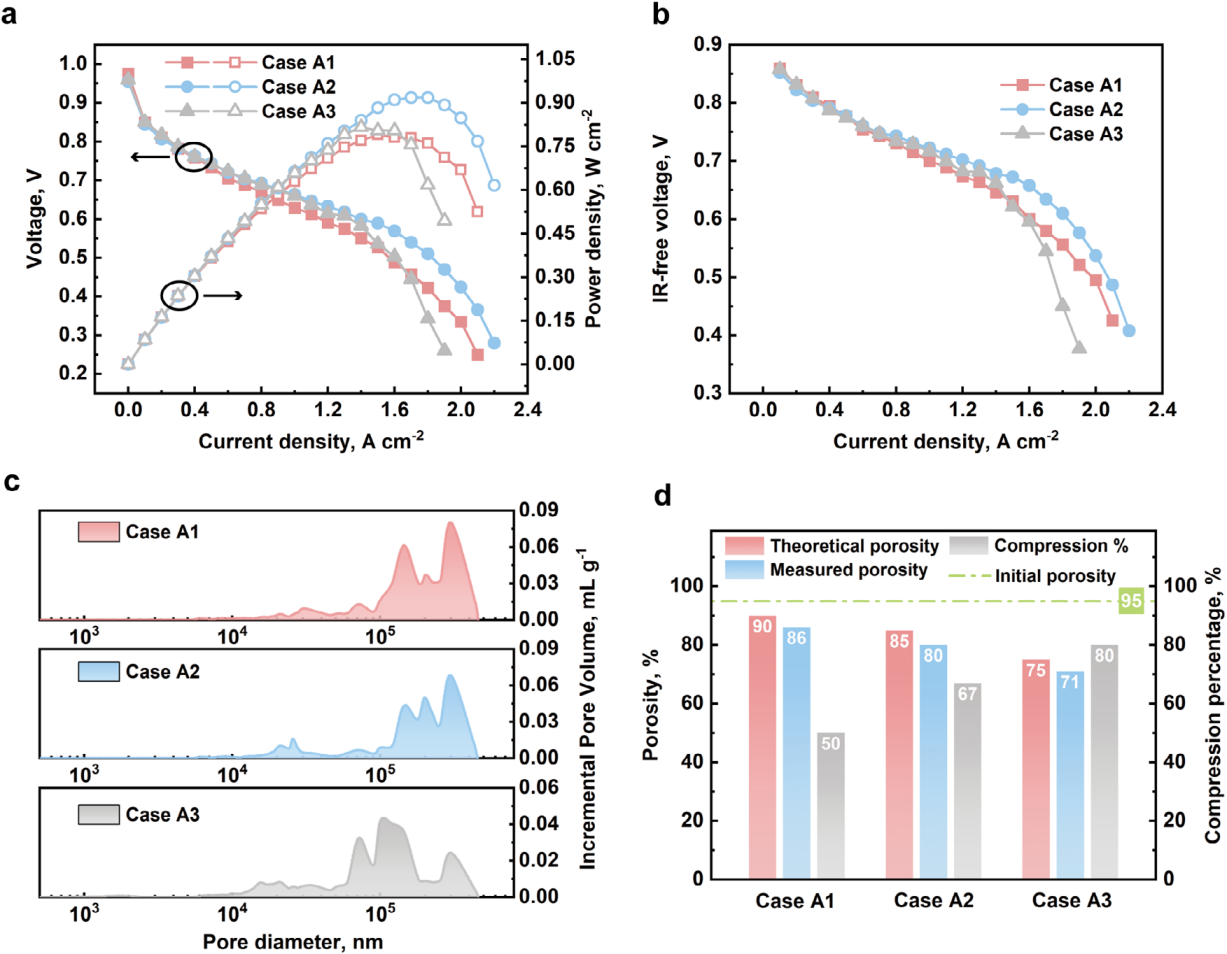
a) The polarization curves and power density curves. Case A1: 50% compression, Case A2: 67% compression, Case A3: 80% compression; b) the IR‐free curves of three cases; c) the MIP measured pore size distribution of all cases; d) the theoretical porosity, compression percentage, MIP measured porosity after compression, and initial porosity of all cases.

Three foam samples with different initial thicknesses, as listed in Table 1, are compressed to 1 mm, assembled, and then tested. The polarization curves and the power density curves of all three cases are shown in **Figure** **3**a. The Ni foam samples with compression percentages of 50%, 67%, and 80% are referred to as case A1, case A2, and case A3, respectively.

Case A2 demonstrates the highest peak power density of 0.918 W cm^−2^, achieved at 0.540 V and 1.7 A cm^−2^. An IR‐free curve represents a voltage‐current profile adjusted to remove the effects of internal resistance (IR) within the electrolyte and cell components. This adjustment is essential in electrochemical studies, as it eliminates the voltage drop caused by ohmic losses, allowing a clearer view of the intrinsic electrochemical behavior. By removing the IR drop, the IR‐free curve provides a more accurate representation of the cell's true performance, highlighting the effects of the electrochemical reaction itself without interference from ohmic losses. The IR‐free curves of all cases are presented in Figure 3b. As shown in figure, case A3 displays more pronounced mass transport losses, which also occur at an earlier stage.

Three foam samples have identical initial width and length, both measuring 5 mm, and a final volume of 25 mm^3^, but differ in their initial thickness. Therefore, the theoretical porosities of three cases are calculated to be 90%, 85%, and 75%, respectively. The final porosity of all samples is measured by means of mercury intrusion porosimetry (MIP). The results of MIP measurement are listed in **Table** **7**. The pore size distribution is shown in Figure 3c. Compression alters the pore size distribution characteristics of the metal foam samples. As shown in Figure 3c, the plot of pore size distribution of case A3 shifts notably to the left compared to the other cases, indicating a reduction in pore size following compression. As shown in Figure 3d, the final porosities of case A1, case A2, and case A3 are 86%, 80%, and 71%, respectively, which are lower than their calculated theoretical value. This discrepancy is likely due to the compression applied, which may have caused the metal foam ligaments to collapse and fold. Such folding can create occluded cavities surrounded by bent ligaments that are inaccessible to fluid under operating pressure, resulting in a porosity value lower than the theoretical calculation. This phenomenon becomes more pronounced with increased compression. As indicated by the threshold pressure values in Table 7 representing the minimum pressure required for mercury to penetrate the smallest pores, the threshold pressure for case A3 is significantly higher than that of the other two cases, which reflects the greater difficulty of fluid penetration due to more extensive collapse and folding of the metal foam ligaments under compression.

**Table 7:** MIP measurement results.

Properties	Case A1	Case A2	Case A3
Total intrusion volume (mL g^−1^)	0.422	0.381	0.328
Average pore diameter (nm):	9.032e4	7.546e4	4.531e4
Porosity (%)	86.061	80.181	71.421
Threshold pressure (psia)	0.83	0.84	1.38
Characteristic length (nm)	2.174e5	2.165e5	1.307e5
Conductivity formation factor	0.521	0.678	0.472
Tortuosity factor	1.258	1.324	1.423

Additionally, compression is known to effectively reduce the electrical resistance of Ni foam. As shown in Table 7, the conductivity formation factor of case A2 is 23% higher than that of case A1, indicating improved conductivity with moderate compression. However, with further compression, the conductivity formation factor of case A3 decreases. This decline suggests that excessive deformation—beyond the foam's yield strength, the maximum stress it can withstand before undergoing irreversible plastic deformation—leads to ligament fractures. This fracturing is caused by the structure reaching a critical stress threshold where it can no longer maintain its integrity, leading to an undesirable increase in resistance.

**Figure 4:**
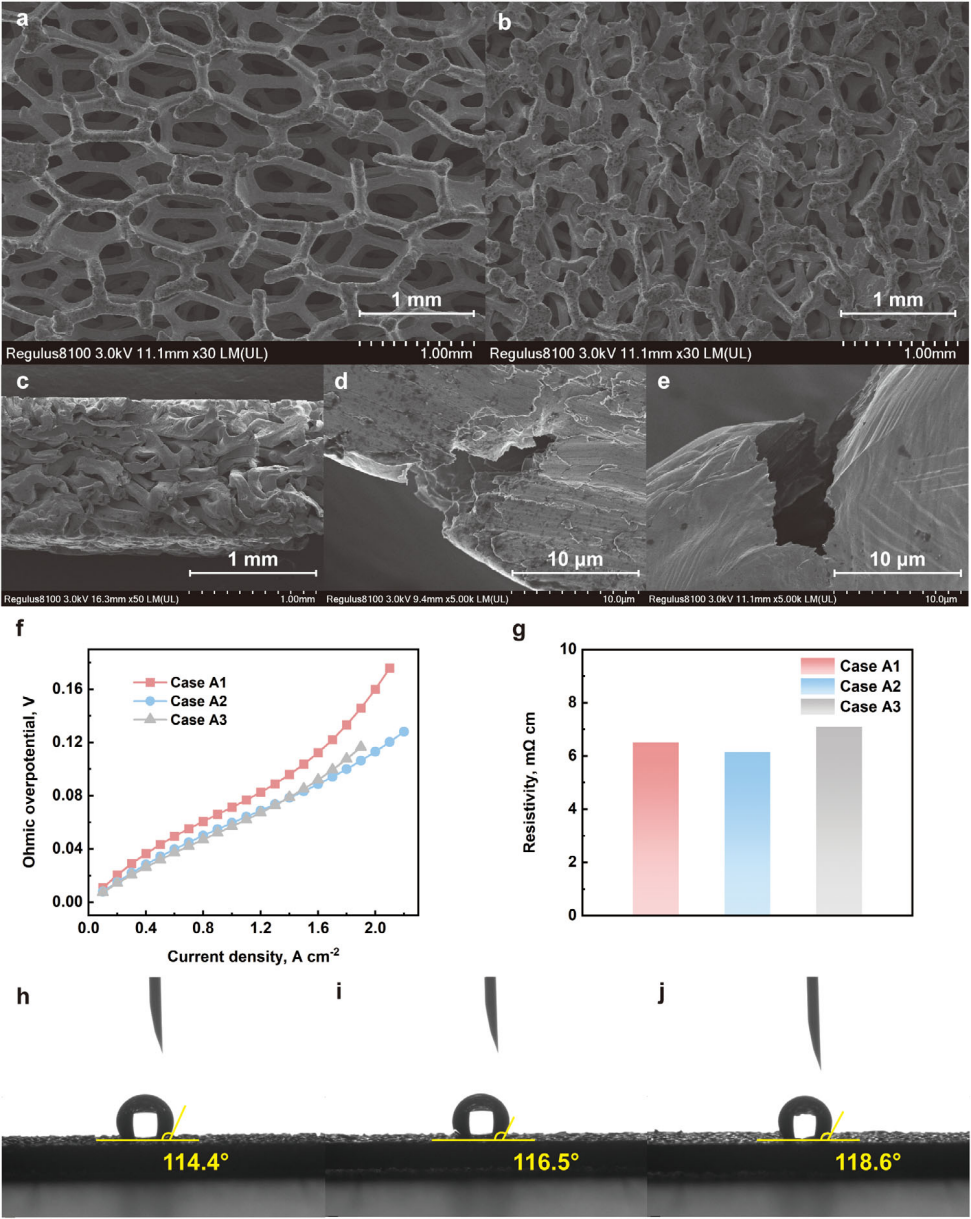
SEM images of Ni foam a) before and b) after compression at 30× magnification; c) the cross‐sectional view of Ni foam with 80% compression percentage; d,e) the foam ligament fracture; f) the ohmic overpotential of all cases; g) the through‐plane resistivity of all cases; h–j) the contact angle of case A1, case A2, and case A3, respectively.

As shown in **Figure** **4**a,b, SEM images of the Ni foam before and after compression at magnifications of 30× reveal distinct occluded cavities regions and fractured ligaments in the compressed foam. Figure 4c shows the cross‐sectional view of case A3 with highly deformed structure. And Figure 4d,e reveals the ligament fractures. Ohmic overpotential is isolated, and as seen in Figure 4f, increasing compression from 50% to 67% effectively reduces ohmic overpotential. However, further increasing compression to 80% results in a slight increase in ohmic overpotential due to fractured ligaments. Additionally, the reduced porosity resulting from occluded cavities created by excessive compression decreases the water management capability of Ni foam, leading to early occurrence of flooding and a rapid voltage drop.

As shown in Figure 4f, the ohmic loss for case A1 exhibits a slightly increasing trend at current densities above 1.6 A cm^−2^. This phenomenon is primarily attributed to two mechanisms. First, since the 50% compression metal foam employed in case A1 possesses better water removal capability, at high current density, the electrochemical reaction rate is increase, so does the electro‐osmotic drag effect, which may result in anode dehydration. Second, our previous simulation study^[^ ^35^ ^]^ demonstrated that PEMFCs using metal foam flow fields, particularly those with higher porosity (such as Case A1), have fewer solid conduction paths, leading to decreased heat dissipation efficiency. The elevated operational temperature further accelerates water evaporation from the membrane. Both mechanisms negatively impact membrane hydration, ultimately decreasing ionic conductivity and increasing ionic ohmic losses.

The resistivity values of all cases are calculated from the results of through‐plane resistance measurement. As shown in Figure 4g, case A2 shows the lowest resistivity (6.15 mΩ cm), while case A3 exhibits the highest resistivity (7.11 mΩ cm) due to ligament fracture, which is consistent with the MIP results.

The contact angle of case A1, case A2, and case A3 are 114.4°, 116.5°, and 118.6°, respectively. As shown in Figure 4h–j, from a macroscopic perspective, increasing the compression percentage can slightly enhance the hydrophobicity of the metal foam.

**Figure 5:**
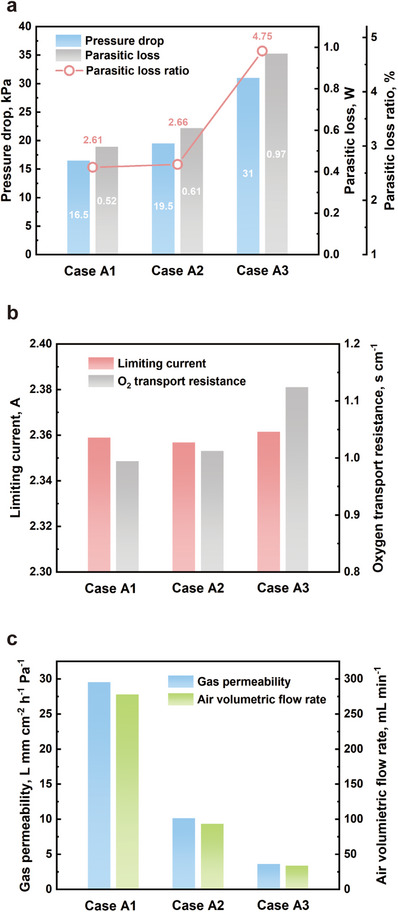
a) the pressure drop, calculated parasitic loss, and parasitic loss ratio of all cases; b) the limiting current and calculated oxygen transport resistance of all cases; c) the in‐plane gas permeability and the measured air volumetric flow rate of all cases.

While a moderate reduction in porosity can enhance output performance by promoting forced through‐plane conduction of reactant toward electrochemical reaction sites and improving water management, an excessive drop in porosity can hinder reactant transport and greatly raise pressure drop, increasing parasitic power losses. The values of pressure drop of the three cases are 16.5 kPa, 19.5 kPa, and 31.0 kPa, respectively, as shown in **Figure** **5**a. Assuming the air compressor has an efficiency of 80%, the calculated parasitic loss and parasitic loss rate are shown in Figure 5a. Increasing the compression percentage from 50% to 67% results in a 6.8% decrease in porosity and a 18.2% increase in pressure drop. However, it also brings a 16% increase in power output with only 0.05% difference in parasitic loss ratio. In contrast, further increasing the compression ratio to 80% significantly boosts the parasitic loss ratio by 82%. Therefore, achieving an optimal balance between the performance gains from enhanced conduction and the potential power losses due to increased pressure drop is essential for maximizing efficiency.

The limiting currents and the calculated oxygen transport resistances for the three cases are shown in Figure 5b. It is evident that excessive compression leads to significant changes in the pore structure of case A3. The oxygen transport resistance of case A3 is significantly higher than that of the other two cases. In addition, in‐plane gas permeability tests are conducted for all three cases, and the results are presented in Figure 5c. It can be observed that the in‐plane permeability of case A3 is only 3.61 mm cm^−2^ h^−1^ Pa^−1^. The impact of compression on in‐plane mass transport in the metal foam flow field is considerably greater than its effect in the through‐plane direction.

**Figure 6:**
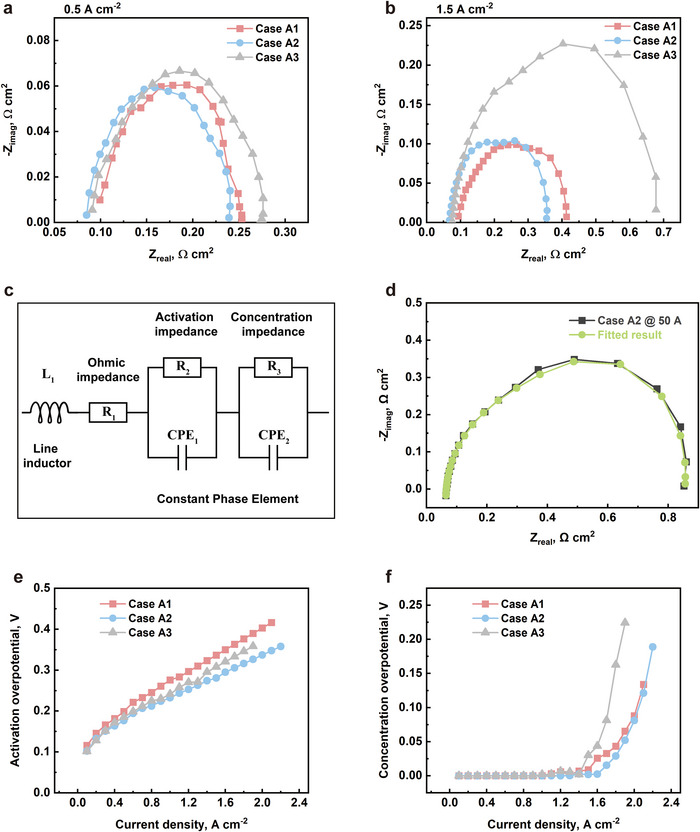
The Nyquist plots of all cases at a) 0.5 A cm^−2^ and b) 1.5 A cm^−2^; c) the equivalent circuit used to fit the EIS results; d) a representative fitting result; e) the activation overpotential and f) the concentration overpotential of all cases.

**Figure** **6**a,b show the EIS results of all cases at low and high current densities. The EIS results are fitted using the equivalent circuit as shown in Figure 6c, with a good fitting achieved, as shown in Figure 6d. Across all current density regions, case A1 exhibits the highest ohmic loss. Part of this ohmic loss is due to ionic ohmic loss. At low current densities, insufficient membrane hydration leads to an increase in ionic ohmic loss. As the current density rises, this issue ameliorates with an increase in membrane water content. Additionally, a decrease in porosity results in greater through‐plane reactant transport, increasing the concentration of reactants at electrochemical reaction sites and allowing more active sites to be utilized. Consequently, as seen in Figure 6e, the activation overpotential of case A1 remains higher than that of the other cases. However, as mentioned previously, excessive compression leading to overly reduced porosity can impair the fuel cell's water management capabilities. This is evident in Figure 6f, where the concentration overpotential of case A3 is significantly higher than that of the other cases.

### 3.2 Pore Size

**Figure 7:**
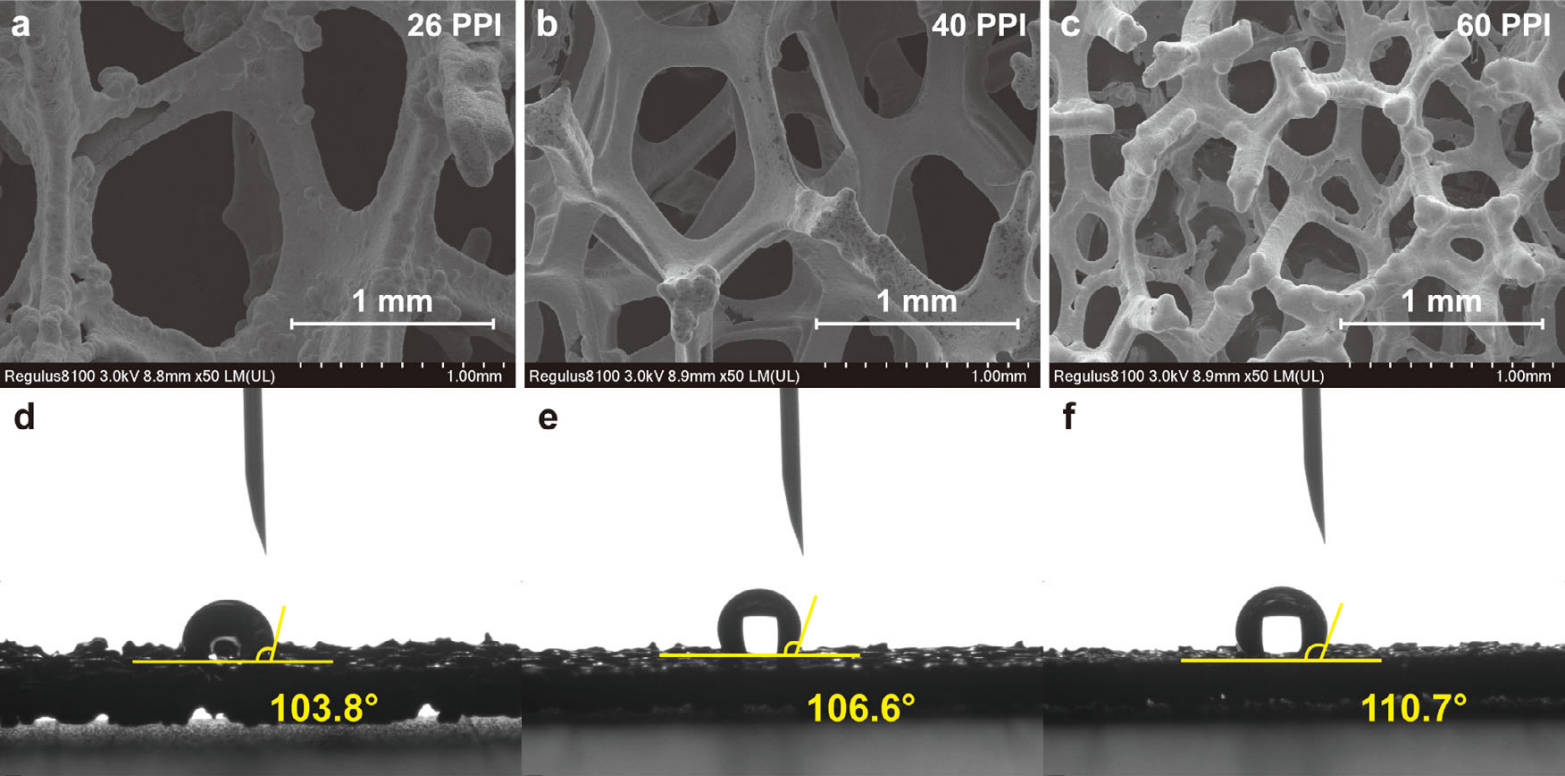
The SEM images of a) case B1 26 PPI; b) case B2 40 PPI; c) case B3 60 PPI, at 50× magnification; the contact angle of d) case B1 26 PPI; e) case B2 40 PPI; f) case B3 60 PPI.

**Figure 8:**
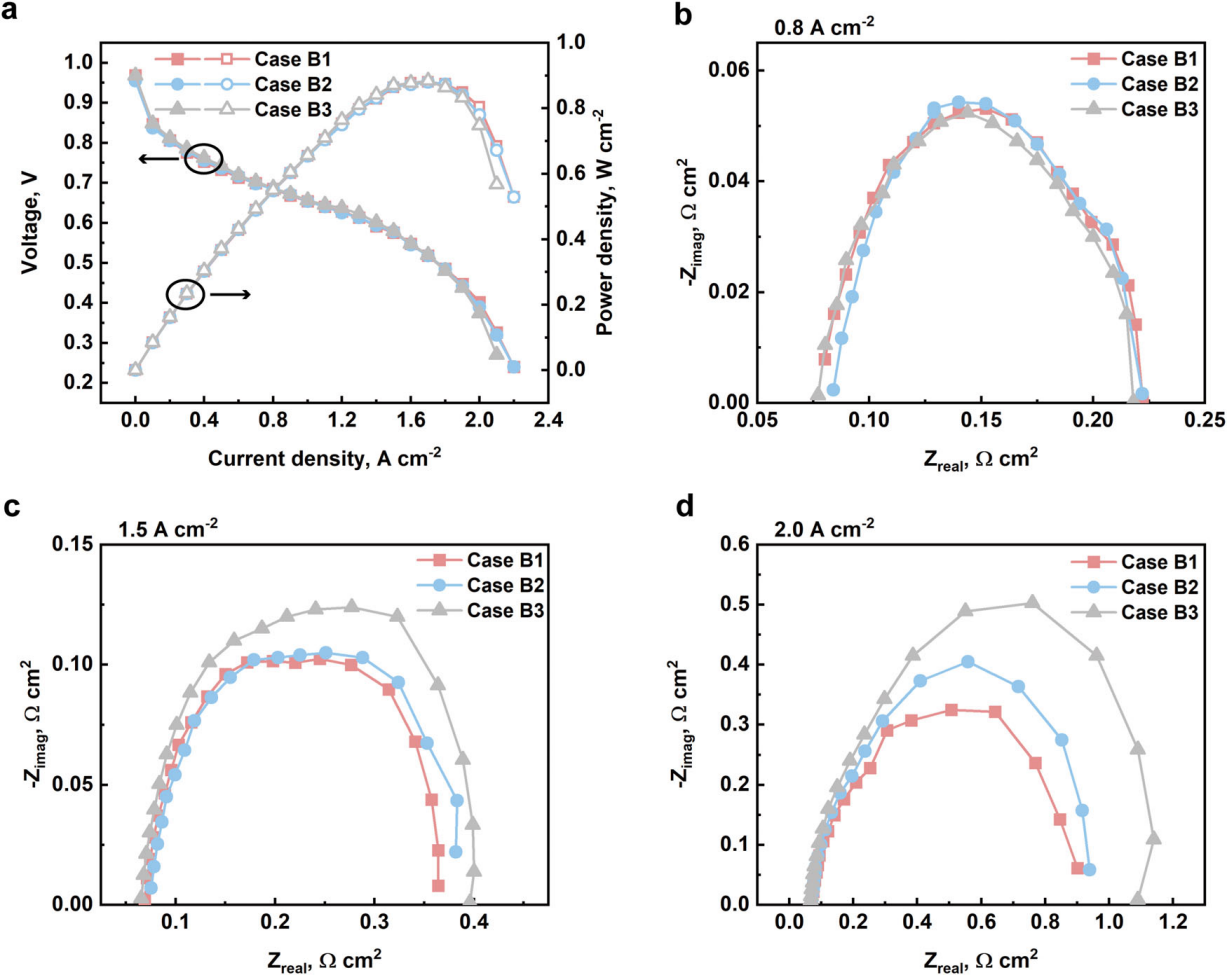
a) The polarization curves and power density curves of case B1: 26 PPI, case B2: 40 PPI, and case B3: 60 PPI; the Nyquist plots of all cases at b) 0.8 A cm^−2^, c) 1.5 A cm^−2^, and d) 2.0 A cm^−2^.

Three foam samples with different pore sizes are compressed from 3  to 1 mm. The Ni foam pore sizes of 26 PPI, 40 PPI, and 60 PPI are denoted as case B1, case B2, and case B3, respectively. The SEM images and the contact angles of all cases are shown in **Figure** **7**a–c,d–f, respectively. The hydrophobicity of metal foam increases as the pore size decreases. The polarization curves and the power density curves of all three cases are shown in **Figure** **8**a. The peak power density of cases B1 and B2 are identical, which are 0.881 W cm^−2^ that achieved at 0.518 V and 1.7 A cm^−2^. The peak power density of case B3 is 0.884 W cm^−2^, achieved at 0.520 V and 1.7 A cm^−2^. The polarization curves of all three cases are nearly identical, except for high current density region, where case B3 starts to drop slightly faster.

The Nyquist plots of each case at various current densities are shown in Figure 8b–d. The Nyquist plots of all cases at 0.8 A cm^−2^ are almost identical. Differences start arising from 1.5 A cm^−2^. At high current density regions, 2.0 A cm^−2^, the ohmic impedances of all cases are still alike. However, the concentration impedance increases as pore size decreases.

**Figure 9:**
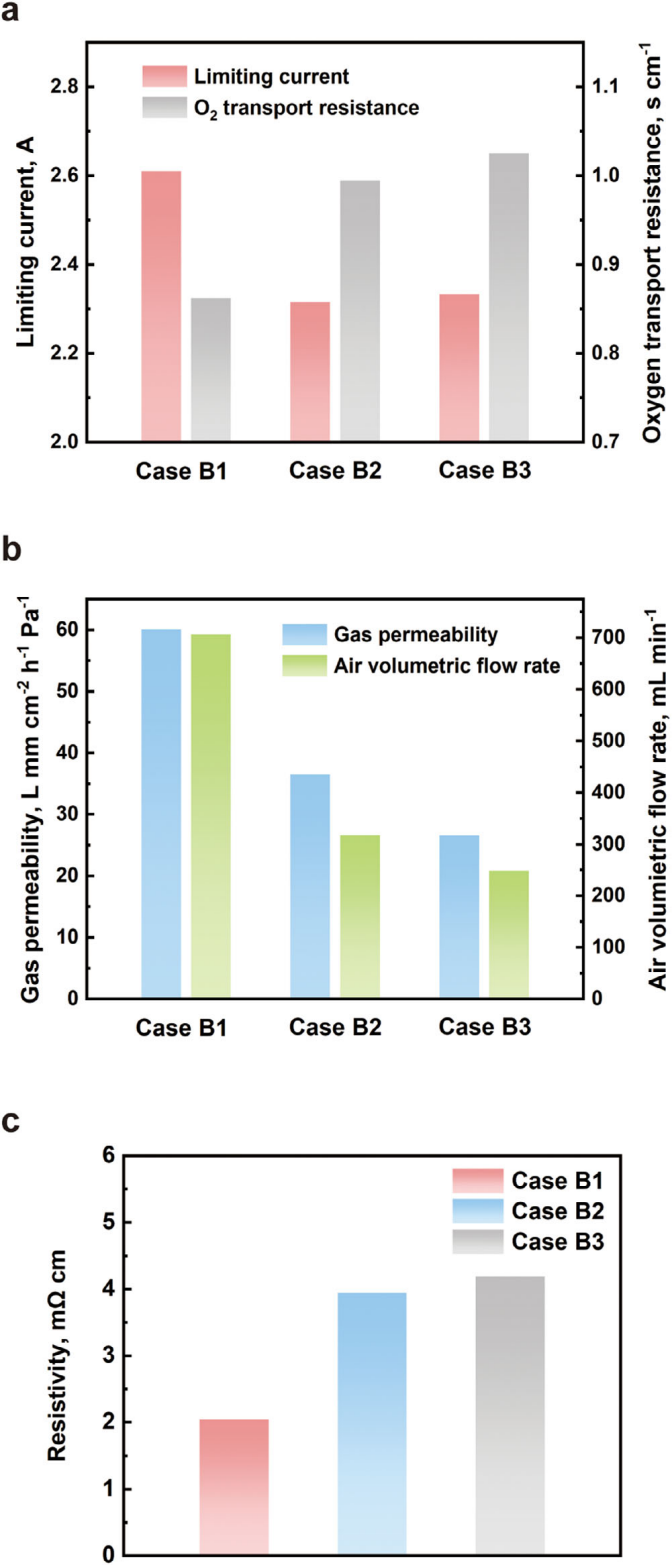
a) the limiting current and calculated oxygen transport resistance of all cases; b) the in‐plane gas permeability and the measured air volumetric flow rate of all cases; c) the through‐plane resistivity of all cases.

In metal foam flow fields with the same porosity, smaller pore sizes typically lead to greater concentration losses due to several interrelated factors. First, metal foams with smaller pores have more ligament walls and longer flow paths. As fluid flows through these smaller pores, it encounters greater resistance, resulting in reduced fluid transport efficiency. This makes it more difficult for reactants to reach the electrochemical reaction sites, thereby impairing mass transfer efficiency and increasing concentration losses. Second, smaller pores create more complex diffusion pathways. With a higher number of small pores per unit volume, gas molecules experience more frequent collisions and tortuous paths during diffusion, which increases diffusion resistance and lowers mass transfer efficiency. As shown in **Figure** **9**a,b, when other structural parameters remain constant, the oxygen transport resistance increases as the pore size decreases. Additionally, in the in‐plane direction, smaller pore sizes can lead to higher flow resistance. Case B3 exhibits the lowest permeability among the three cases, with a value of only 26.58 mm cm^−2^ h^−1^ Pa^−1^.

Third, the removal of liquid water becomes more challenging in structures with smaller pores. In fuel cells, water generation is inevitable, and smaller pores are more prone to retaining liquid water, leading to pore blockage that further impedes gas flow and aggravates concentration losses. In contrast, larger pore structures are more effective at removing liquid water, reducing the risk of pore blockage or flooding. Therefore, metal foams with smaller pore sizes exhibit higher concentration losses due to greater fluid resistance, more complex diffusion paths, and an increased risk of water blockage.

However, despite the disadvantages in mass transport, smaller pores have the potential to enhance heat dissipation. The increased ligament density and greater solid surface area of smaller pores facilitate improved thermal conductivity and heat transfer. This suggests that smaller pore sizes may provide advantages in heat management, though this comes at the expense of gas transport efficiency. On the other hand, as shown in Figure 7, under approximately same porosity, metal foams with larger pore sizes tend to have thicker ligaments compared to those with smaller pore sizes. This characteristic may make large‐pore foams less favorable for thermal management; however, the thicker ligaments are more beneficial for electronic conduction. The through‐plane resistivities of three cases are plotted in Figure 9c, where case A1 exhibits a significantly lower resistivity than the other cases. Therefore, balancing pore size to optimize both thermal management and mass transport remains a critical design consideration for PEMFCs.

### 3.3 Hydrophobicity

51.32 g of 5 wt.% PTFE dispersion is prepared by mixing 4.28 g of 60 wt.% PTFE dispersion with 47.04 g of deionized water, and ultrasonically dispersed at 53 kHz under 30 °C for 10 min. Three 5 × 5 cm^2^ pristine Ni foams are hand‐cut, compressed air blown, ethanol cleaned, then oscillated using the ultrasonic cleaner at 53 kHz for 3 min to remove any debris or dirt on their surface. After vacuum drying, the samples are weighed and their initial weights are recorded as m_i_. The foam samples are dipped into the 5 wt.% PTFE dispersion and agitated for 5 s, then put into the vacuum drying machine that is preheated to 80 °C. After drying, their weights with PTFE loaded are recorded as m_d_. The two‐step heat treatment is conducted, strictly following the procedure as described in Section 2.1.3. The final weights of the samples are recorded as m_f_. Weight loss and the final PTFE loading are calculated as listed in **Table** **8**. Then three foam samples with different PTFE loading are compressed from 3 mm to 1 mm using the universal test machine, and 30 MPa is applied.

**Table 8:** Properties of all Ni foam samples used in PTFE loading investigation.

Property	Case C1	Case C2	Case C3
m_i_	0.9051 g	0.8747 g	0.8933 g
m_d_	0.9266 g	0.9272 g	0.9992 g
m_f_	0.9255 g	0.9246 g	0.9937 g
Weight loss	5.1%	4.9%	5.2%
PTFE loading	2.2 wt.%	5.4 wt.%	10.1 wt.%

**Figure 10:**
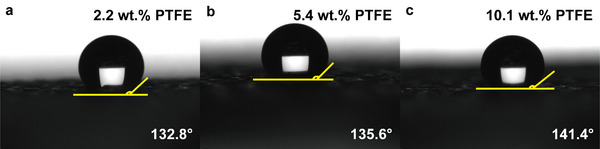
Results of contact angle measurement: a) 2.2 wt.% PTFE loading; b) 5.4 wt.% PTFE loading; and c) 10.1 wt.% PTFE loading.

The Ni foam with PTFE loading of 2.2 wt.%, 5.4 wt.%, and 10.1 wt.% are denoted as case C1, case C2, and case C3, respectively. The contact angles of case C1, case C2, and case C3 are 132.8°, 135.6°, and 141.4°, as shown in **Figure** **10**a–c, respectively. From Figure 10, the hydrophobicity of Ni foam is proportional to its PTFE loading. Higher PTFE loading results in higher hydrophobicity, as expected.

**Figure 11:**
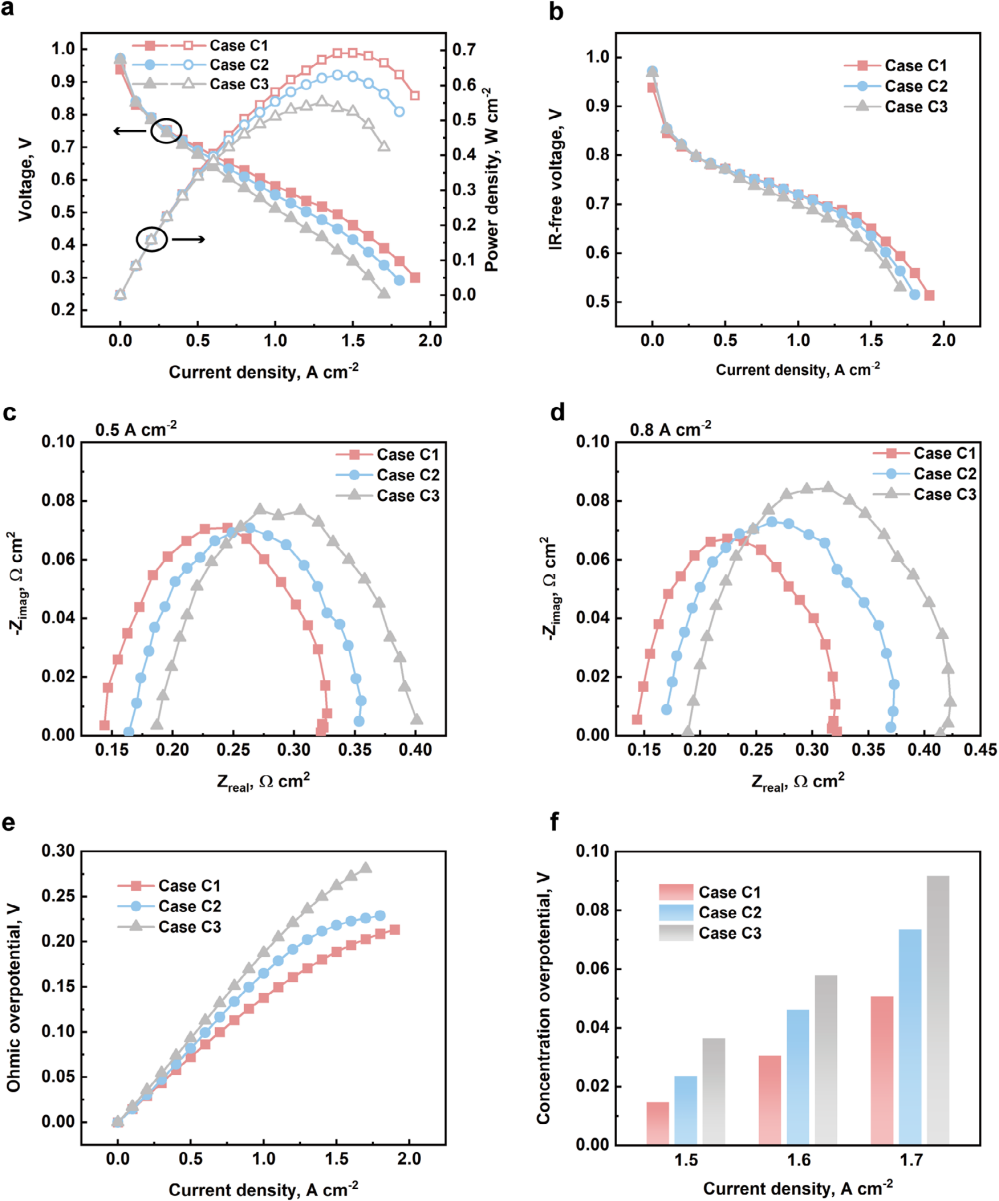
a) The polarization curves and power density curves of Ni foam with different PTFE loading, case C1: 2.2 wt.%, case C2: 5.4 wt.%, case C3: 10.1 wt.%; b) the IR‐free curves for all cases; the Nyquist plots of all cases at c) 0.5 A cm^−2^ and d) 0.8 A cm^−2^; e) the ohmic overpotential and f) concentration overpotential of all cases.

The polarization curves and the power density curves of all three cases are shown in **Figure** **11**a. Case C1 achieved the highest peak power density of 0.693 W cm^−2^ that measured at 0.462 V and 1.5 A cm^−2^. From the polarization curves, the major difference among the three cases is the ohmic overpotential, which is impacting the performance of PEMFC directly in all current density regions. The IR‐free curves and ohmic overpotential curves are shown in Figure 11b,e. PTFE coating is often used for surface modification, making the surface hydrophobic. Additionally, PTFE has exceptional chemical stability, providing strong resistance to chemical corrosion. This allows metal foam to achieve better durability in the high‐humidity and acidic environment of PEMFCs. However, PTFE is an insulating material with high electrical resistivity. Therefore, the Ni foam flow field with higher PTFE loading possesses greater electrical resistance.

The Nyquist plots for each case at various current densities are shown in Figure 11c,d. The case with higher PTFE loading exhibits greater ohmic impedance, consistent with the observations from the polarization curves. Starting from 0.8 A cm^−2^, all cases begin to display two distinguishable arcs, with the concentration arc appearing earlier. Figure 11e presents the fitted ohmic overpotential, where it is evident that the ohmic overpotential increases with higher PTFE loading. Across the entire current density range, the ohmic overpotential of case C3 remains higher than that of the other cases.

**Figure 12:**
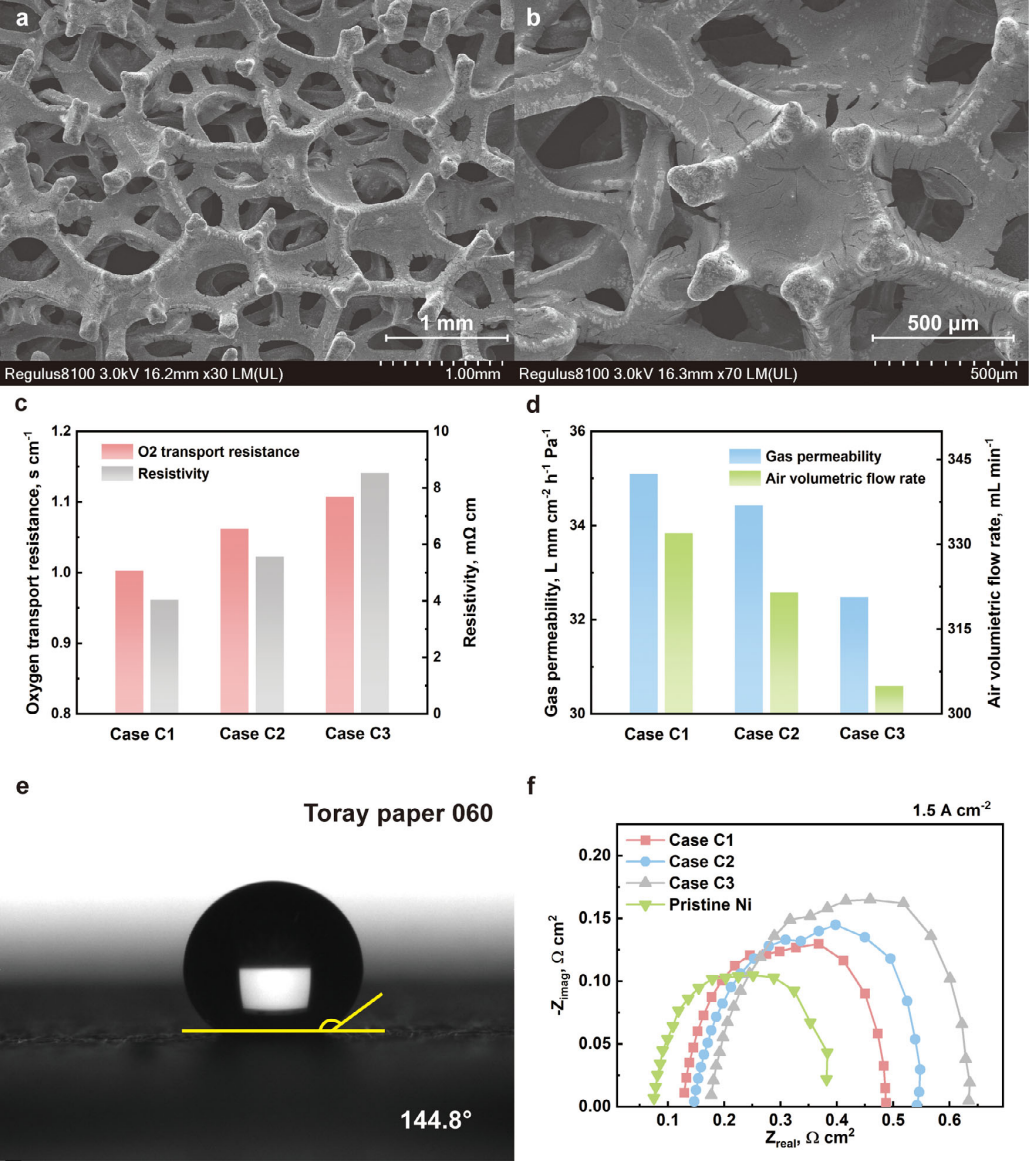
SEM images of PTFE coated Ni foam at a) 30× and b) 70× magnification; c) the oxygen transport resistance and through‐plane resistivity of all cases; d) the in‐plane gas permeability and measured air volumetric flow rate of all cases; e) result of contact angle measurementof Toray paper 060; f) comparison of Nyquist plots of case C1, case C2, case C3, and pristine Ni at 1.5 A cm^−2^.

Figure 11f shows the concentration overpotential for the three cases at high current densities, which exhibits a similar trend to the ohmic overpotential. As hydrophobicity increases, the concentration overpotential also rises. Theoretically, PTFE coating can provide corrosion resistance for Ni foam and make the environment in the flow field hydrophobic, which is easier for water inside the flow field to be expelled from it. However, the surface hydrophobicity may repel water from leaving GDL and entering the flow field. The higher the hydrophobicity is, the stronger the repulsion is. Hence, case C3 shows the largest activation impedance and concentration impedance, which is believed that resulted from flooding at the electrode. Additionally, excessive PTFE loading increases the possibility of pores being blocked by PTFE, which hinders mass transport and reduces water management capabilities. This eventually results in higher concentration losses. As shown in the **Figure** **12**a,b, plenty pores are partially or completely blocked. Additionally, to quantify the impact of excessive PTFE on mass transport, limiting current measurements and permeability measurements are conducted, and the results are shown in Figure 12c,d. Mass transport in both the in‐plane and through‐plane directions clearly deteriorates with increasing PTFE loading. This is primarily because, as the loading increases, more pores become partially or completely blocked.

Moreover, a comparison is made by adding a PEMFC utilizing uncoated pristine Ni foam as the cathode flow field into consideration. The pristine Ni foam is much less hydrophobic than other PTFE‐treated samples. For reference, the contact angle of a commercial MPL‐coated Toray 060 carbon paper, which is used as the GDL in these experiments, is also measured, as shown in Figure 12e. The surface of Toray 060 paper is the most hydrophobic among all tested samples. As shown in Figure 12f, at 1.5 A cm^−2^, the pristine Ni foam exhibits the lowest ohmic, activation, and concentration impedances. This suggests that a less hydrophobic or even hydrophilic surface is beneficial for metal foam cathode flow fields in PEMFCs, as it promotes better water removal from the electrode.

### 3.4 Surface Treatment

In this section, a new 5 × 5 cm^2^ pristine Ni foam is prepared for PTFE coating. The initial weight m_i_ is 0.5976 g. After dipping into the 5 wt.% PTFE dispersion and drying, the dry weight is recorded as m_d_ = 0.6319 g. After the heat treatment, the final weight of the sample is recorded as m_f_ = 0.6304 g. Weight loss is ≈4.4%. The final PTFE loading is 5.2 wt.%.

**Figure 13:**
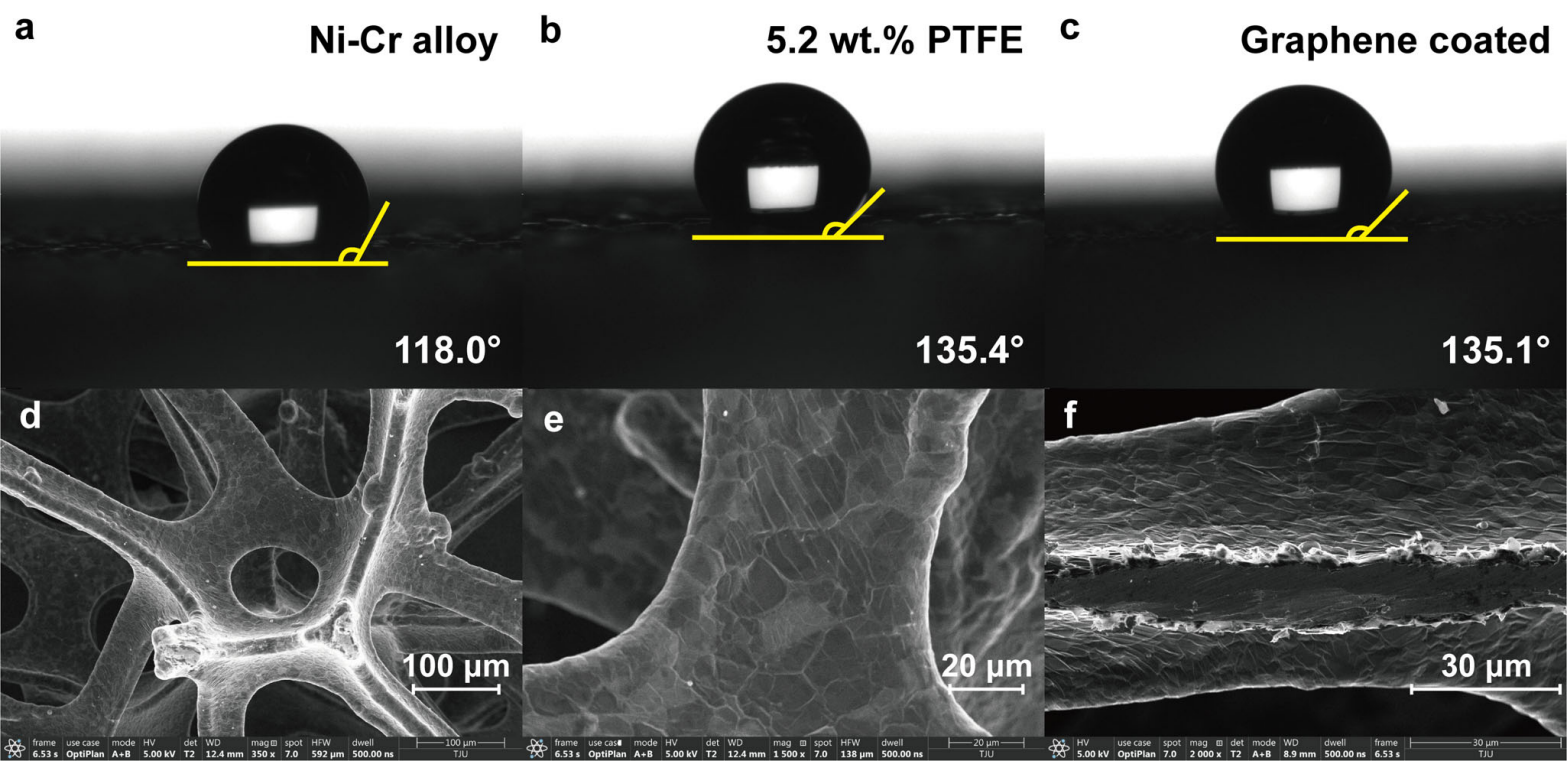
Results of contact angle measurement of a) Ni‐Cr alloy foam; b) PTFE coated Ni foam with 5.2 wt.% PTFE loading; and c) graphene‐coated Ni foam;^[^ ^33^ ^]^ SEM images of graphene coating at d) 350 × and e) 1500 × magnification; f) the cross‐sectional SEM image of graphene coating at 2000 × magnification.

Contact angle measurements of Ni‐Cr alloy foam, 5.2 wt. % PTFE‐coated Ni foam, and graphene coated Ni foam are performed and the results are 118°, 135.4°, and 135.1°,^[^ ^33^ ^]^ as shown in **Figure** **13**a–c, respectively. The graphene coated Ni foam and 5.2 wt.% PTFE coated Ni foam have approximate hydrophobicity and the Ni‐Cr alloy foam possesses the lowest hydrophobicity. The SEM images of the graphene‐coated Ni foam surface at magnifications of 350×, 1500×, and the cross‐section at 2000× are shown in Figure 13d–f, respectively. The SEM images reveal a uniform graphene coating distributed across the surface of the Ni foam.

**Figure 14:**
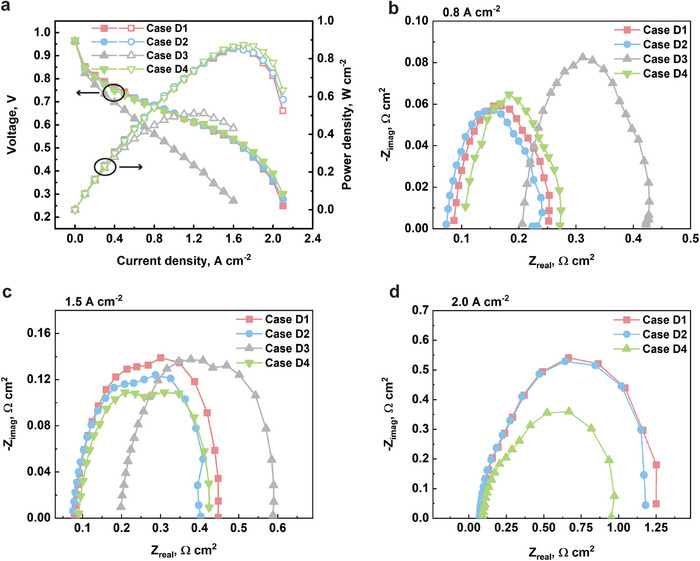
a) The polarization curves and power density curves of case D1: Ni‐Cr alloy foam, case D2: pristine Ni foam, case D3: 5.2 wt.% PTFE‐coated Ni foam, and case D4: graphene‐coated Ni foam; the Nyquist plots of all cases at b) 0.8 A cm^−2^, c) 1.5 A cm^−2^, and d) 2.0 A cm^−2^.

The polarization curves and the power density curves of all four cases are shown in **Figure** **14**a. The Ni‐Cr alloy foam, the pristine Ni foam, the 5.2 wt.% PTFE‐coated Ni foam, and the graphene‐coated Ni foam are denoted as case D1‐D4, respectively. Case D4 achieves the highest peak power density of 0.874 W cm⁻^2^ at 0.514 V and 1.7 A cm⁻^2^. Cases D1 and D2 exhibit similar performance, with peak power densities of 0.853 W cm⁻^2^ and 0.856 W cm⁻^2^, respectively. In contrast, case D3 demonstrates the lowest peak power density of 0.511 W cm⁻^2^. From the polarization curves, case D1, D2, and D4 show comparable performance at low and moderate current density regions, while case D4 displays slightly better performance after the current density exceeds 1.6 A cm^−2^. The polarization curve of case D3 indicates a significant ohmic overpotential.

The Nyquist plots comparing the four cases at various current densities are shown in Figure 14b–d. At 0.8 and 1.5 A cm^−2^, as seen in Figure 14b,c, case D1, D2, and D4 have comparable activation and ohmic impedances, while the ohmic impedance of case D3 is significantly greater than those of the other cases. This large ohmic impedance in case D3 is attributed to its PTFE coating, which is an electrically insulative material. In contrast, the graphene coating demonstrates superior performance. At 2.0 A cm^−2^, where the electrochemical reaction rate is higher and more water is produced, case D4 continues to maintain relatively lower concentration impedance compared to cases with uncoated foam, as shown in Figure 14d. At limiting current density, the voltage of case D4 is 21% and 9% higher than that of case D1 and case D2, respectively, exhibiting an outstanding capability of water management.

When comparing the Nyquist plots of case D3 and case D4 at 1.5 A cm^−2^, despite both cases having similar contact angles, case D4 still has a lower concentration impedance. This can be attributed to the smaller pore size of 110 PPI, which makes it easier for the PTFE solution to block internal cells of the metal foam during the coating process. The blocked cells no longer provide effective pathways for gas or water transport, complicating water management and ultimately leading to increased concentration impedance.

**Figure 15:**
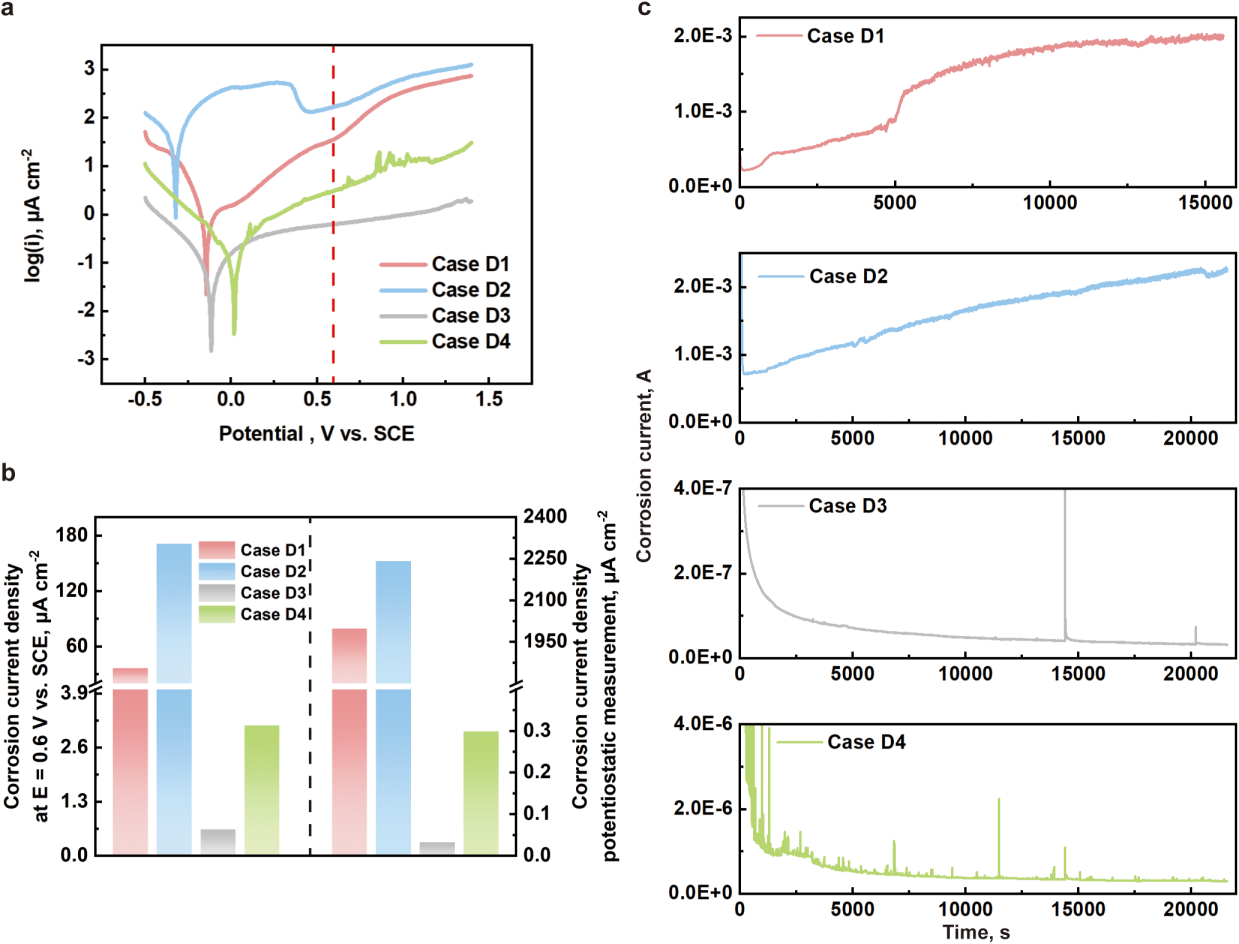
a) The potentiodynamic test results of all cases; b) the corrosion current density at E = 0.6 V versus SCE and the average corrosion current density of last 300‐s data from potentiostatic tests of all cases; c) the potentiostatic test results of all cases.

The coating not only serves to alter the hydrophobicity of the metal foam surface for improved water management but also provides sufficient corrosion resistance for nickel foam in the acidic, high‐temperature, and high‐humidity environment of PEMFCs. To investigate this, four circle samples measuring 1 cm^2^ in area and 0.5 mm in thickness are prepared using Ni‐Cr alloy, pristine nickel plate, PTFE coated Ni plate, and graphene coated Ni plate, respectively. Potentiodynamic measurement is performed to characterize the relationship between corrosion potential and corrosion current. Potentiostatic measurement is also conducted to evaluate the anti‐corrosive capability of each sample under long‐term operating conditions. The results are shown in **Figure** **15**. As shown in Figure 15a, from the polarization curves measured from potentiodynamic test, both the PTFE and graphene coatings, as well as the Ni‐Cr alloy, exhibit significantly lower corrosion current density compared to pristine nickel under conditions simulating the operating environment of a proton exchange membrane fuel cell. The corrosion current density at 0.6 V versus SCE is used for comparison. As shown on the left side of Figure 15b, the corrosion current densities of the PTFE‐coated sample (case D3) and the graphene‐coated sample (case D4) are 0.636 µA cm^−2^ and 3.132 µA cm^−2^, respectively. In contrast, the pristine Ni sample (case D2) exhibits a significantly higher corrosion current density of 171 µA cm^−2^. The Ni‐Cr alloy provides a certain degree of corrosion protection, but its corrosion current density still reaches 36.48 µA cm^−2^, which is much higher than that of the PTFE‐ and graphene‐coated samples.

The results of the potentiostatic test are plotted in Figure 15c. For case D1 and case D2, their corrosion current densities exceeded the milliampere level ≈5000 s into the test, which is unacceptable. In contrast, both case D3 and case D4 maintained a consistently low corrosion current density throughout the entire 21600‐s potentiostatic test. As shown on the right side of Figure 15b, the corrosion current density of the PTFE‐coated sample is 0.0324 µA cm^−2^, while the graphene‐coated sample is 0.299 µA cm^−2^. Both coatings provide effective corrosion resistance for nickel under PEMFC operating conditions. However, considering the electrical resistance associated with the PTFE coating, the graphene coating appears to be a more favorable choice. It provides sufficient hydrophobicity and corrosion resistance while maintaining excellent electrical conductivity.

## 4 Conclusion

This study comprehensively examines the effects of structural and surface modifications of nickel foam on its performance as a cathode flow field in PEMFCs. The porosity, pore size, hydrophobicity, and surface coatings are identified as critical factors influencing electrochemical performance, water management, and corrosion resistance.

The porosity is controlled by altering the compression percentage, which significantly affects both structural integrity and electrochemical properties. A moderate compression percentage of 67% optimizes electrical conductivity and mass transport, achieving a conductivity formation factor 23% higher than the uncompressed sample. However, excessive compression results in ligament fractures and occluded cavities, increasing resistance and harming water management capabilities. This also alters the pore size distribution, leading to reduced pore sizes, early flooding, and rapid voltage drops at high current densities. Furthermore, actual porosity consistently falls below theoretical values due to occluded cavities, as evidenced by higher threshold pressures, higher oxygen transport resistance, and lower gas permeability in highly compressed samples. While moderate porosity reductions enhance forced reactant through‐plane transport and water removal, excessive reductions lead to unwanted increases in pressure drop and parasitic power losses, emphasizing the need to balance compression and porosity for optimal performance.

Pore size has more pronounced impact at high current densities. Smaller pores increase concentration losses due to higher fluid resistance, more complex diffusion pathways, and greater water retention, which hinder mass transfer efficiency. Larger pores, on the other hand, effectively mitigate water retention and pore blockage, improving mass transport and water management. Despite these drawbacks, smaller pores offer potential advantages in heat dissipation due to their higher ligament density and greater surface area, which enhance thermal conductivity. While, larger pore is beneficial for electrical conductivity. Additional, smaller pore can provide a more uniform surface contact with components. Balancing pore size to optimize mass transport and thermal management remains a key design consideration.

While higher PTFE loading increases hydrophobicity, it also raises ohmic losses due to PTFE's insulating properties, with excessive hydrophobicity hindering water transfer from the GDL to the flow field. Additionally, excessive PTFE loading may block pores, impairing mass transport and water management. Comparisons with pristine Ni foam and Toray 060 GDL demonstrate that moderate hydrophobicity improves water removal, suggesting an optimal hydrophobicity to balance corrosion resistance, water management, and electrical conductivity for peak performance.

Lastly, surface coatings are evaluated for their effects on corrosion resistance and electrochemical performance. While all coatings effectively reduce corrosion current density compared to pristine nickel, graphene‐coated Ni foam achieves the best overall performance. It demonstrates superior water management, the highest peak power density, and excellent electrical conductivity, particularly at high current densities. These results position graphene as a promising coating for durable and efficient metal foam flow fields in PEMFCs, balancing hydrophobicity, corrosion resistance, and conductivity. Additionally, the porosity and pore size of metal foam directly affect the complexity of its internal structure and the surface area exposed to the corrosive environment. Higher porosity and smaller pore size generally result in a larger specific surface area, which can increase the material's susceptibility to corrosion in the acidic, high‐temperature, and high‐humidity conditions of PEMFCs. Moreover, enhancing the surface hydrophobicity of metal foam helps reduce water retention on the material's surface, thereby shortening the exposure time to corrosive agents and improving corrosion resistance. Therefore, increasing hydrophobicity is considered an effective strategy for improving the durability of metal foam in PEMFC applications. By optimizing these parameters, the corrosion resistance of metal foam flow fields can be improved.

In summary, this study highlights the interconnected influence of structural and surface properties on PEMFC performance and emphasizes the importance of multi‐factor optimization to achieve durable and efficient nickel foam flow fields for practical applications. Future research should further optimize the interaction among metal foam structural parameters to enhance performance. Hybrid coatings that combine materials like graphene and other alloy should be explored to balance hydrophobicity, corrosion resistance, and conductivity. Dynamic water and thermal management strategies, such as designing gradients in hydrophobicity or tailored pore structure, should be investigated to improve mass transport and heat dissipation. Additionally, long‐term durability testing under real‐world conditions and cost‐effective, scalable fabrication methods are critical for transitioning these innovations into practical applications.

## Conflict of Interest

The authors declare no conflict of interest.

## Author Contributions

S.W. performed conceptualization, data curation, formal analysis, investigation, methodology, validation, visualization, acquired funding acquisition, wrote the original draft. C.T. performed data curation, investigation, validation, visualization, wrote reviewed and edited the final manuscript. X.R. performed data curation, investigation, wrote reviewed and edited the final manuscript. Q.D. performed conceptualization, formal analysis, methodology, project administration, resources, supervision, validation, wrote reviewed and edited the final manuscript. J.W.P. performed conceptualization, formal analysis, investigation, methodology, supervision, validation, wrote reviewed and edited the final manuscript. K.J. performed formal analysis, methodology, project administration, resources, supervision, validation, acquired funding acquisition, wrote reviewed and edited the final manuscript.

## Data Availability

The data that support the findings of this study are available from the corresponding author upon reasonable request.

## References

[1] K. Jiao , B. Wang , Q. Du , Y. Wang , G. Zhang , Z. Yang , H. Deng , X. Xie , Water and Thermal Management of Proton Exchange Membrane Fuel Cells, Elsevier, Amsterdam, New York 2021.

[2] B. Wang , Z. Yang , M. Ji , J. Shan , M. Ni , Z. Hou , J. Cai , X. Gu , X. Yuan , Z. Gong , Q. Du , Y. Yin , K. Jiao , Energy and AI 2023, 14, 100278.

[3] Y. Chen , J. Zhang , S. Zhai , Z. Hu , Energy and AI 2024, 16, 100345.

[4] T. Suzuki , A. Iiyama , N. Kubo , N. Saito , K. Shinohara , S. Shimotori , Y. Sugawara , K. Yamada , ECS Trans. 2019, 92, 3.

[5] Y. Wang , K. Wu , H. Zhao , J. Li , X. Sheng , Y. Yin , Q. Du , B. Zu , L. Han , K. Jiao , Energy and AI 2023, 11, 100205.

[6] S. T. Bunyan , H. A. Dhahad , D. S. Khudhur , T. Yusaf , Sustainability 2023, 15, 10389.

[7] B. H. Lim , E. H. Majlan , W. R. W. Daud , T. Husaini , M. I. Rosli , Ionics 2016, 22, 301. 10.1016/j.heliyon.2018.e00845 PMC6190529 30338304

[8] W. Huo , B. Xie , S. Wu , L. Wu , G. Zhang , H. Zhang , Z. Qin , Y. Zhu , R. Wang , K. Jiao , Int. J. Green Energy. 2024, 21, 154.

[9] Z. Wang , Z. Liu , L. Fan , Q. Du , K. Jiao , Energy Reviews 2023, 2, 100017

[10] B. Wang , W. Chen , F. Pan , S. Wu , G. Zhang , J. W. Park , B. Xie , Y. Yin , K. Jiao , J. Power Sources. 2019, 434, 226741.

[11] Y. Pang , Y. Wang , Energy and AI 2023, 14, 100265.

[12] Q. Ding , H.‐L. Zhao , Z.‐M. Wan , Y.‐R.u Yang , C. Yang , X.‐D. Wang , J. Energy Eng. 2020, 146, 04020054.

[13] X.‐D. Wang , X.‐X. Zhang , W.‐M. Yan , D.‐J. Lee , A.y Su , Int. J. Hydrogen Energy. 2009, 34, 3823.

[14] A. P. Manso , F. F. Marzo , J. Barranco , X. Garikano , M. Garmendia Mujika , Int. J. Hydrogen Energy. 2012, 37, 15256.

[15] S. Shimpalee , S. Greenway , J. Van Zee , J. Power Sources. 2006, 160, 398.

[16] L. Lin , X. Zhang , H. Feng , X. Wang , Sci. China Technol. Sci. 2010, 53, 453.

[17] H. Ashrafi , N. Pourmahmoud , I. Mirzaee , N. Ahmadi , Iran. J. Chem. Chem. Eng. 2023, 42, 1.

[18] Y. Kerkoub , A. Benzaoui , F. Haddad , Y. K. Ziari , Energy Convers. Manage. 2018, 174, 260.

[19] Z. Wang , L. Fan , S. Wu , C. Tongsh , Y. Zhang , Z. Yang , Q. Du , D. Hao , F. Zhou , K. Jiao , Front. Thermal Eng. 2022, 2, 900910.

[20] L. Chen , Z. Wang , C. Sun , H. Zhu , Y. Xia , G. Hu , B. Fang , Micromachines 2023, 14, 1224. 37374810 10.3390/mi14061224 PMC10304778

[21] Z. Li , F. Bai , P. He , Z. Zhang , W.‐Q. Tao , Int. J. Hydrogen Energy 2023, 48, 27778.

[22] A. A. Aly , S. J. Abideen , Y. Tao , D. Ø. Madsen , High Temp. Mater. Processe. 2023, 42, 20220290.

[23] W. C. Tan , L. H. Saw , H. S. Thiam , J. Xuan , Z. Cai , M. C. Yew , Renewable Sustainable Energy Rev. 2018, 96, 181.

[24] W. Yuan , Y. Tang , X. Yang , Z. Wan , Appl. Energy 2012, 94, 309.

[25] G. Zhang , Z. Qu , W.‐Q. Tao , X. Wang , L. Wu , S. Wu , X.u Xie , C. Tongsh , W. Huo , Z. Bao , K. Jiao , Y. Wang , Chem. Rev. 2022, 123, 989. 36580359 10.1021/acs.chemrev.2c00539

[26] D. G. Kang , C. Park , D. K. Lee , D. K. Shin , M. S. Kim , Int. J. Hydrogen Energy. 2024, 60, 611.

[27] G. Zhang , Z. Bao , B. Xie , Y. Wang , K. Jiao , Int. J. Hydrogen Energy. 2021, 46, 2978.

[28] Q. Li , K. Sun , M. Suo , Z. Zeng , C. Guan , H. Liu , Z. Che , T. Wang , Appl. Energy. 2024, 365, 123273.

[29] S. Huo , L. Li , W. Shi , R. Wang , B. Lu , Y. Yin , C. Zhu , Y. Wang , K. Jiao , Z. Hou , Int. J. Green Energy. 2021, 18, 1129.

[30] S. Huo , N. J. Cooper , T. L. Smith , J. W. Park , K. Jiao , Appl. Energy. 2017, 203, 101.

[31] S. Wu , C. Tongsh , Q. Du , J. W. Park , K. Jiao , Int. J. Green Energy. 2025, 1, 10.1080/15435075.2024.2446505.

[32] S. Huo , W. Shi , R. Wang , B. Lu , Y. Wang , K. Jiao , Z. Hou , Sci. China Technol. Sci. 2021, 64, 1041.

[33] C. Tongsh , S. Wu , K. Jiao , W. Huo , Q. Du , J. W. Park , J. Xuan , H. Wang , N. P. Brandon , M. D. Guiver , Joule 2024, 8, 175.

[34] K.‐Y. Law , J. Phys. Chem. Lett. 2014, 5, 686. 26270837 10.1021/jz402762h

[35] W. Huo , S. Wu , Z. Bao , C. Tongsh , B. Xie , M. Benbouzid , F. Gao , Y. Amirat , K. Jiao , eTransportation 2025, 24, 100398.

